# Arbuscular mycorrhizal fungi community analysis revealed the significant impact of arsenic in antimony- and arsenic-contaminated soil in three Guizhou regions

**DOI:** 10.3389/fmicb.2023.1189400

**Published:** 2023-05-18

**Authors:** Yidong Mi, Chao Xu, Xinru Li, Min Zhou, Ke Cao, Cuimin Dong, Xuemei Li, Ningning Ji, Fanfan Wang, Hailei Su, Xuesong Liu, Yuan Wei

**Affiliations:** ^1^State Key Laboratory of Environmental Criteria and Risk Assessment, Chinese Research Academy of Environmental Sciences, Beijing, China; ^2^College of Environment, Hohai University, Nanjing, China; ^3^Chinese Research Academy of Environmental Sciences, Beijing, China; ^4^College of Environmental Science and Engineering, Tongji University, Shanghai, China

**Keywords:** arbuscular mycorrhizal fungi, antimony, arsenic, bioremediation, community composition

## Abstract

**Introduction:**

The lack of systematic investigations of arbuscular mycorrhizal fungi (AMF) community composition is an obstacle to AMF biotechnological applications in antimony (Sb)- and arsenic (As)-polluted soil.

**Methods:**

Morphological and molecular identification were applied to study the AMF community composition in Sb- and As-contaminated areas, and the main influencing factors of AMF community composition in Sb- and As-contaminated areas were explored.

**Results:**

(1) A total of 513,546 sequences were obtained, and the majority belonged to Glomeraceae [88.27%, 193 operational taxonomic units (OTUs)], followed by Diversisporaceae, Paraglomeraceae, Acaulosporaceae, Gigasporaceae, and Archaeosporaceae; (2) the affinity between AMF and plants was mainly related to plant species (*F* = 3.488, *p* = 0.022 < 0.050), which was not significantly correlated with the total Sb (TSb) and total As (TAs) in soil; (3) the AMF spore density was mainly related to the available nitrogen, available potassium, and total organic carbon; (4) The effect of soil nutrients on AMF community composition (total explanation: 15.36%) was greater than that of soil Sb and As content (total explanation: 5.80%); (5) the effect of TAs on AMF community composition (λ = −0.96) was more drastic than that of TSb (λ = −0.21), and the effect of As on AMF community composition was exacerbated by the interaction between As and phosphorus in the soil; and (6) Diversisporaceae was positively correlated with the TSb and TAs.

**Discussion:**

The potential impact of As on the effective application of mycorrhizal technology should be further considered when applied to the ecological restoration of Sb- and As-contaminated areas.

## 1. Introduction

Antimony (Sb) and arsenic (As) have similar chemical characteristics (Anawar et al., 2011; Yu et al., 2021) and occur ubiquitously in the environment at trace levels (Johnston et al., 2020; Xu et al., 2020; Li Y. et al., 2021). Both Sb and As have been listed as priority pollutants by the European Union and the Environmental Protection Agency of the USA and restricted pollutants by China (Wei et al., 2015a; Chang et al., 2022; Yang et al., 2022; Zhou et al., 2022). Sb is carcinogenic and excessive Sb exposure can damage the respiratory, cardiovascular, and urinary systems (Nishad and Bhaskarapillai, 2021; Liu et al., 2022; Zhou et al., 2022). China is the world’s largest producer of Sb, especially southwest China, which accounts for 80% of global Sb production (Xu et al., 2020). Sb is typically accompanied by As in sulfide ores in Sb mining areas, which are found worldwide (Li et al., 2017; Chang et al., 2022). As is also a metalloid and a primary environmental pollutant and carcinogen (Li B. et al., 2021; Ozturk et al., 2022; Wu et al., 2022). The Qinglong Sb mine is a typical Sb mining area in southwest China, where the concentrations of soil Sb and As reach as high as 5,447 and 472 mg/kg, respectively, which are much higher than the Chinese background soil value of Chinese values (Mao et al., 2022), which has affected the residents living near the Qinglong Sb mine. The consumption of contaminated foods or drinking water was found to be the dominant dietary intake source of Sb and As for the residents near an Sb mining area, for whom the total dietary intakes were 554 and 306 μg/day, respectively (Wu et al., 2011; Chang et al., 2022). Reducing Sb and As exposure and the subsequent harm to the human body urgently need to be addressed.

There are a variety of microorganisms in the ecosystem that can mutualism with plants and play a role in the resistance of plants to biotic and abiotic stresses (Xiao et al., 2022; Yan et al., 2022). Arbuscular mycorrhizal fungi (AMF) are ubiquitous in soil environments and interact with most plants through mycorrhizal structures (Xiao et al., 2022). The application of AMF to enhance plant resistance to heavy metal toxicity and improve phytoremediation efficiency is an ecologically friendly soil remediation technology that has been widely researched (Gu et al., 2017; Bi et al., 2019; Riaz et al., 2021). Currently, there are two common strategies for the application of AMF to reduce human exposure to Sb or As. The first strategy is to use AMF to enhance the Sb and As enrichment of plants (Wei et al., 2016; Liu et al., 2020) or to improve the Sb or As tolerance of plants (Zhang and Chen, 2021; Xi et al., 2022), so as to reduce the content or toxicity of Sb or As in soil, and finally realize the ecological restoration of Sb- or As-polluted areas. Another strategy is to use AMF to reduce Sb or As migration into agricultural products or to convert Sb or As into less toxic forms to reduce human exposure to Sb or As (Alam et al., 2019; Li et al., 2022). Therefore, the rational application of AMF has great significance for ecological restoration and food security around Sb- and As-contaminated areas.

Considering the key role of AMF in soil ecosystems, it is important to understand the community composition and diversity distribution of AMF in Sb- and As-contaminated areas and the effects of Sb and As on AMF for ecological restoration and food security around Sb- and As-contaminated areas. Wei et al. (2015a) investigated the AMF of a typical Sb mining area in China and concluded that Sb contamination was the dominating factor influencing the AMF community composition in the Sb mining area. However, this study ignored the potential effect of As in the Sb mining area. Schneider et al. (2013) studied AMF communities in an As-contaminated area of Brazil, and they found that As contamination reduced the AMF species richness, but the effects of other soil factors on the AMF community composition were not systematically discussed. Parvin et al. (2019) conducted a detailed analysis of the effects of As pollution on the AMF community composition in paddy fields in Bangladesh. However, the results of this study have little significance for the application of AMF in the ecological restoration of Sb and As mining areas. The lack of research on AMF community composition and its influencing factors in Sb- and As-co-contaminated areas is an obstacle to the effective implementation of ecological remediation technology in Sb- and As-contaminated areas.

The Qinglong Sb mine in Guizhou Province is one of the main Sb mining areas in China, and it has been exploited for nearly 60 years. Historical mining and smelting activities in the Qinglong Sb mine have caused serious Sb and As pollution that threatens the health of nearby residents (Liu et al., 2011). The preliminary survey found that AMF was widely distributed in this area, thus making the study area an ideal system for the evaluation of AMF community composition in Sb- and As-contaminated soil systems. In this study, the AMF community composition was characterized using high-throughput sequencing technology in three sites with different Sb and As concentrations. The study aimed to (1) identify the factors affecting the AMF-plant symbiosis in Sb- and As-contaminated areas and explore the effects of Sb and As pollution on the AMF colonization rate and spore density; (2) investigate the relationship between the soil properties and the AMF community composition and identify the main factors affecting the AMF community composition in Sb- and As-contaminated areas; and (3) screen the dominant AMF in Sb- and As-contaminated areas for ecological restoration in Sb- and As-contaminated areas.

## 2. Materials and methods

### 2.1. Study area and soil sampling

Samples were collected in the Qinglong Sb mining area, which is situated in Qinglong County, Qianxinan Buyei and Miao Autonomous Prefecture, Guizhou Province, China. This area belongs to a karst landform with abundant groundwater, and has a plateau subtropical climate, with a mean annual temperature of 14.1°C and mean annual rainfall of 1,500 mm.

**FIGURE 1:**
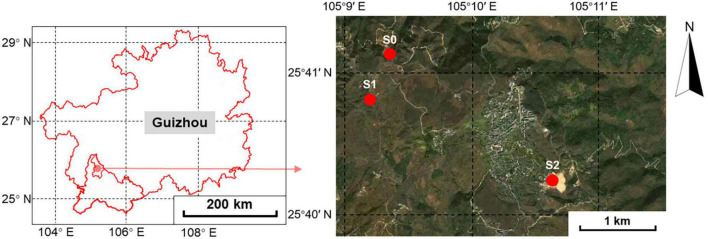
Geographic location of the sampling sites in Qinglong County, Qianxinan Buyei and Miao Autonomous Prefecture, Guizhou Province.

Samples were collected in August 2021. Based on the preliminary investigation, three sampling sites were selected according to the mining function, namely S1, S2, and S0. S1 was the smelting area with a total Sb (TSb) of 3,800–27,300 mg/kg and total As (TAs) of 193–1,160 mg/kg. S2 was the tailings pond with a TSb of 2,640–48,000 mg/kg and TAs of 1,090–2,980 mg/kg. The undisturbed site S0 was located over 1 km away from the mining area. The sampling point information is shown in Figure 1 and Supplementary Table 1. The TSb of S0 (405.89 mg/kg) was slightly higher than the second type of land control value limit specified in the risk control standard for soil contamination of development land of China (360 mg/kg) (Ministry of Ecology and Environment of the People’s Republic of China, 2018), and the TAs of S0 (85.03 mg/kg) was lower than the first type of land control value limit specified in the risk control standard for the soil contamination of development land of China (120 mg/kg) (Ministry of Ecology and Environment of the People’s Republic of China, 2018). Four 20 m × 20 m plots were selected at S1 and S2, and three plots were selected at S0. Dominant plant and 1 kg rhizosphere soil samples were collected from the topsoil (0–10 cm) associated with *Artemisia argyi* (Aa), *Rumex acetosa* (Ra), *Boehmeria nivea* (Bn), *Buddleja lindleyana* (Bl), and *Carpesium abrotanoides* (Ca). Three well-grown individuals of each plant species were collected from each plot and mixed into one sample. The dominant plants selected at each sampling site are shown in Supplementary Table 1. Each sample was then divided into two subsamples, with one refrigerated in liquid nitrogen and sent immediately to the laboratory for molecular analysis and the other was used to analyze the spore density and soil properties after air-drying. The roots of plants were used to measure the AMF colonization rate.

### 2.2. Measurement of soil properties, AMF colonization rate, and spore density

The alkali N-proliferation method was used to measure available nitrogen (AN). Briefly, 2 g of air-dried soil was alkalized with 10 ml 1.8 mol/L NaOH solution and the alkalized products were absorbed with 3 ml 20 g/L H_3_BO_3_ indicator and titrated with 0.01 mol/L HCl solution. Extraction with NH_4_F-HCl solution (pH < 6.5) or NaHCO_3_ solution (pH > 6.5) and estimation using the molybdenum-antimony colorimetric method was utilized to determine available phosphorus (AP). The total calcium (TCa) was measured with an inductively coupled plasma optical emission spectrometer (ICP-OES, 5110, Agilent Technologies, Santa Clara, CA, USA), and the available potassium (AK) was estimated with the ammonium acetate method and flame photometry (AA-6880, Shimadzu, Shanghai, China). The soil total organic carbon (TOC) was measured using a TOC analyzer (Vario TOC Select, Elementar, Beijing, China). The soil pH and electrical conductivity (EC) were estimated with a pH meter (FE28, Mettler Toledo, Shanghai, China) after adding 25 ml of water to 10 g of air-dried soil.

The TSb and TAs were extracted by adding 6 ml of aqua regia (1.5 ml HCl and 4.5 ml HNO_3_) to 0.1 g dry soil sample and were digested using a microwave digestion system (Mars 6 Classic 910980, CEM, Charlotte, NC, USA). The digestion procedure consisted of heating for 5 min and maintaining a temperature of 120°C for 2 min; heating for 4 min and maintaining 150°C for 5 min; and heating for 5 min and maintaining 185°C for 40 min. The extract was filtered through a 0.45-μm nylon filter, diluted to 50 ml with pure water, and analyzed using inductively coupled plasma mass spectrometry (ICP-MS, 7900, Agilent Technologies, Santa Clara, CA, USA). The diethylenetriamine pentaacetic acid (DTPA)-extractable Sb (DTPA-Sb) and DTPA-extractable As (DTPA-As) concentrations were determined by ICP-OES (5110, Agilent Technologies, Santa Clara, CA, USA) after a 10 g sample was dissolved using DTPA extraction agent [DTPA (0.005 M), CaCl_2_ (0.01 M), and triethanolamine (TEA, 0.1 M, pH 7.3)]. All of the soil data are shown in Table 1.

**TABLE 1:** Physicochemical properties of the sampling sites.

Sampling site	AN (mg/kg)	AP (mg/kg)	AK (mg/kg)	TOC (g/kg)
S0	155.56 ± 28.44^b^	1.35 ± 0.25^b^	250.22 ± 58.64^b^	30.867 ± 6.981^b^
S1	343.17 ± 103.35^a^	8.50 ± 12.04^b^	398.17 ± 133.48^a^	74.438 ± 54.580^a^
S2	39.73 ± 22.23^c^	64.00 ± 25.23^a^	182.33 ± 47.15^b^	16.138 ± 22.759^b^
**Sampling site**	**TSb (g/kg)**	**DTPA-Sb (mg/kg)**	**TAs (g/kg)**	**DTPA-As (mg/kg)**
S0	0.406 ± 0.153^b^	0.25 ± 0.23^b^	0.085 ± 0.014^c^	0.26 ± 0.18
S1	12.311 ± 7.255^a^	10.85 ± 14.56^ab^	0.614 ± 0.347^b^	0.35 ± 0.28
S2	20.053 ± 14.370^a^	16.83 ± 14.58^a^	2.103 ± 0.681^a^	0.30 ± 0.24
**Sampling site**	**pH**	**EC (mS/m)**	**TCa (g/kg)**	**–**
S0	7.18 ± 0.37	5.14 ± 3.50^b^	8.797 ± 2.639^c^	–
S1	7.36 ± 0.61	9.46 ± 3.05^b^	33.858 ± 26.351^b^	–
S2	7.29 ± 0.50	128.69 ± 46.25^a^	59.242 ± 18.478^a^	–

Different lowercase letters indicate significant differences at *p* < 0.05.

The AMF colonization rates were measured according to Vierheilig et al. (2005). In brief, roots were cleaned and cut into 2-cm segments. Then the root segments were transferred to 10% potassium hydroxide (KOH) and heated in a 90°C water bath for 1 h. The root segments were stained with 5% ink-vinegar for 5 min, cleaned with tap water that contained several drops of acetic acid, and observed under a microscope (NSZ-810, Beijing East-Bio Technology Co. Ltd., Beijing, China). Spores were isolated from approximately 20 g of a soil sample via wet sieving, decanting, and centrifugation (3,000 rpm, 2 min) through a 60% (weight/volume) sucrose cushion. Spores were counted using a microscope (NSZ-810, Beijing East-Bio Technology Co. Ltd., Beijing, China).

### 2.3. DNA extraction, polymerase chain reaction (PCR) amplification, and sequencing

Total DNA was extracted from 0.25 g soil with an E.Z.N.A.^®^ soil DNA Kit (Omega Bio-Tek, Beijing, China) according to the manufacturer’s instructions. The 18S rRNA of AMF was subjected to two-step nested PCR. The PCR program and reaction system components are shown in Supplementary Table 2. AML1: 5′-ATCAACTTTCGATGGTAGGATAGA-3′ and AML2: 5′-GAACCCAAACACTTTGGTTTCC-3′ were used as primers in the first-round PCR, and AMV4.5NF: 5′-AAGCTCGTAGTTGAATTTCG-3′ and AMDGR: 5′-CCCAACTATCCCTATTAATCAT-3′ were used as primers in the second-round PCR. The PCR products were purified using an AxyPrep DNA Gel Extraction Kit (Axygen Biosciences, Union City, CA, USA) and quantified using QuantiFluor-ST (Promega, Beijing, China). Purified amplicons were sequenced using an Illumina MiSeq platform (Majorbio, Shanghai, China) via 2 × 250 bp paired-end sequencing. Raw reads have been submitted to the NCBI sequence read archive (SRA) database with the following accession number: PRJNA943530.

Sequences were demultiplexed as fastq files and quality-filtered using a QIIME pipeline (version 1.17) (Xiao B. et al., 2019). Sequences were clustered into operational taxonomic units (OTUs) at 97% sequence similarity, and the chimeric sequences were removed using the UPARSE program (version 7.1).^1^ The taxonomic classification of each 18S rRNA gene sequence was analyzed with the Ribosomal Database Project classifier^2^ against the MaarjAM 18S rRNA database (Amato et al., 2013).

### 2.4. Statistical and data analysis

Data analyses were performed with the SPSS 17.0 software package. Differences between the means of soil properties were assessed using analysis of variance (ANOVA) tests, and the Duncan test was used to compare the homogeneity of variance at the 0.05 probability level (*p* = 0.05). The observed richness (Sobs), Shannon, Shannoneven, and Coverage indexes were calculated using Mothur (Version 1.80.2).^3^ The AMF colonization rate, spore density, and the Sobs, Shannon, Shannoneven, and Coverage index box plots were constructed using R, and the significance of the differences were tested using an ANOVA test. The significance levels (F values) of the effects of plants, sampling sites, and their interactions on the AMF colonization rate, spore density, and AMF community index were tested using multi-way ANOVA analysis. The AMF community bar plot at the family level was plotted with R. Principal coordinates analysis (PCoA) was employed to illustrate AMF community composition clustering using the R package based on the Bray–Curtis distance. Permutational multivariate ANOVA (PERMANOVA) and analysis of similarities (ANOSIM) based on the Bray–Curtis distance were conducted to analyze the significant difference in the AMF community compositions among the sampling sites. The rarefaction curve was performed using R. Heat trees were generated using the log2 ratios of the median counts for each taxon and the Wilcox rank-sum test followed by a Benjamini and Hochberg correction for multiple testing using the “Metacoder” package in R (Foster et al., 2016). The variance inflation factor (VIF) was calculated in SPSS 17.0 and used to diagnose multicollinearity, environmental factors with VIF > 10 were screened for canonical correlation analysis (CCA) analysis (Guzman et al., 2021). The correlation between soil properties and AMF communities was elucidated through CCA using the R package. Variation partitioning analysis (VPA) was conducted in R using the “vegan” package to partition the influences of soil nutrients, heavy metals, and pH and TCa on AMF community composition. A Mantel test was used to compare the correlations between soil properties and the AMF colonization rate, spore density, and the Sobs, Shannon, and Shannoneven indexes, and between soil properties and AMF families in R using the “ggcor” package. The correlations between the soil properties and the AMF colonization rate, spore density, and the Sobs, Shannon, and Shannoneven indexes were obtained based on the Pearson correlation coefficient. Two-factor correlation network analysis was performed using Gephi 0.9.7. The heatmaps were created using the R package and the correlations were obtained based on the Pearson correlation coefficient. To estimate the direct and indirect effects of the soil properties on the AMF richness and diversity and spore density, a structural equation model (SEM) was fitted to the data using AMOS (IBM SPSS AMOS 26). The soil nutrients were synthetic variables derived from the first principal components generated by the principal component analysis (PCA) of AN, AK, and TOC (total explainable variance = 81.5%); the AMF richness and diversity were synthetic variables derived from the first principal components generated through PCA of the Sobs, Shannon, and the Shannoneven indexes (total explainable variance = 86.5%).

## 3. Results

### 3.1. AMF colonization rate of plant and AMF spore density in soil

**FIGURE 2:**
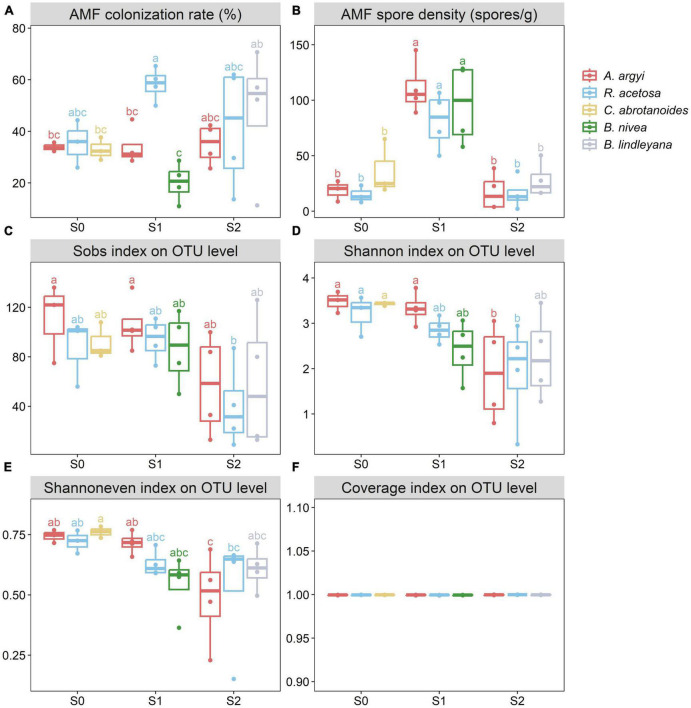
Arbuscular mycorrhizal fungi (AMF) colonization rate **(A)**, spore density **(B)**, and the Sobs **(C)**, Shannon **(D)**, Shannoneven **(E)**, and Coverage **(F)** indexes of different plants in different sampling sites. The line in the box represents the median value, box boundaries indicate values in the 25–75th percentile range, and whiskers indicate the 95% confidence intervals. Different letters indicate significant differences at *p* < 0.05. The absence of the same letter labels between any two boxes indicates that there is a significant difference.

The AMF infection structures in different plants are shown in Supplementary Figure 1. AMF within *A. argyi* roots produced numerous external hyphae, and vesicles formed at the tips of the external hyphae. AMF associated with *R. acetosa* and *C. abrotanoides* roots produced numerous internal hyphae and vesicles. AMF within *B. nivea* roots produced twining hyphae without vesicles. *R. acetosa* and *B. lindleyana* had relatively high AMF colonization rates (Figure 2A). There were significant differences in the colonization rates among S1 plants, with S1_Ra (sampling site_plant species) detecting the highest colonization rate (58.25 ± 6.41%). However, there were no significant differences among the sampling sites. The influence of different plant species (*F* = 3.488, *p* = 0.022 < 0.050) on the AMF colonization rate was greater than that of different sampling sites (*F* = 1.301, *p* = 0.291) (Supplementary Table 3).

There were 15 to 111 spores/g soil in the rhizosphere of different plants in different sampling sites (Figure 2B). The spore density in the rhizosphere soil of S1 was significantly higher than those of S0 and S2. The influence of different sampling sites (*F* = 33.978, *p* < 0.001) on the spore density was greater than that of different plant species (*F* = 0.982, *p* = 0.436) (Supplementary Table 3).

### 3.2. Overall sequencing results and AMF community composition

DNA was extracted from the 33 soil samples and amplified via PCR using the AML1 and AML2 primers in the first round and the AMV4.5NF and AMDGR primers in the second round. All of the reads assigned to AMF were aligned against the AMF sequences in the MaarjAM database. After leveling out according to the minimum number of sample sequences, 513,546 sequences were obtained. Overall, 268 AMF OTUs were detected, and a greater number of OTUs belonged to the Glomeraceae (88.27%, 193 OTUs, 453,321 sequences). Others belonged to the Diversisporaceae (6.09%, 16 OTUs, 31,293 sequences), Paraglomeraceae (3.81%, eight OTUs, 19,548 sequences), and Acaulosporaceae (1.47%, 14 OTUs, 7,571 sequences), while only a small number belonged to Gigasporaceae (0.22%, seven OTUs, 1,127 sequences) and Archaeosporaceae (0.03%, 11 OTUs, 160 sequences). In addition, 0.10% of all of the sequences were unclassified at the family level. Detailed information on the OTUs in each sampling site is provided in Supplementary Table 4. The AMF community composition at the family level across different plants from the three investigated sampling sites was explored (Supplementary Figure 2). Glomeraceae was the dominant family in all of the groups of samples, and the relative abundance of each sample ranged from 78.41% in S1_Ra to 96.39% in S0_Ra.

The β-diversity of the AMF communities across different sampling sites and different plants based on the Bray–Curtis distance (Supplementary Figure 3). The total explanatory degree of the first two principal components of AMF community composition was 27.38%. The distribution of points in S0 was the most concentrated, and S2 was the most dispersed. The influence of different plants and different sampling sites on the AMF community was analyzed using PERMANOVA (Supplementary Table 5). The results showed that different plants (*R*^2^ = 0.163, *p* = 0.028 < 0.05) and different sampling sites (*R*^2^ = 0.189, *p* < 0.001) significantly affected the AMF community. The AMF communities between S0 and S1, S0 and S2, and S1 and S2 showed significant dissimilarities, which was confirmed by ANOSIM based on the Bray–Curtis distance (*p* < 0.001) (Supplementary Table 5). Among them, S1 and S2 had the lowest dissimilarity, followed by S0 and S1 (Supplementary Figure 3 and Supplementary Table 5).

The AMF richness, diversity, evenness, and coverage on the OTU level among the different sampling sites were estimated using the Sobs, Shannon, Shannoneven, and Coverage indexes (Figures 2C–F). Most rarefaction curves tended to saturate (Supplementary Figure 4). As shown in Figure 2, the Sobs index of different sampling sites indicated no significant differences in the richness of S0 and S1, but they were both higher than those of S2, and S0_Aa and S1_Aa had significantly higher richness than S2_Ra. The order of the Shannon index values among the three sampling sites was S0 > S1 > S2, and the diversity of S0 and S1_Aa was significantly higher than S2_Aa and S2_Ra. The Shannoneven index of S0 was higher than that of S1 and S2. The influence of different sampling sites (*F* = 6.041, *p* = 0.007 < 0.010 for Sobs; *F* = 8.198, *p* = 0.002 < 0.010 for Shannon; *F* = 5.987, *p* = 0.008 < 0.010 for Shannoneven) on the AMF richness, diversity, and evenness was greater than that of different plant species (*F* = 0.593, *p* = 0.671 for Sobs; *F* = 0.912, *p* = 0.468 for Shannon; *F* = 1.153, *p* = 0.356 for Shannoneven) (Supplementary Table 3).

**FIGURE 3:**
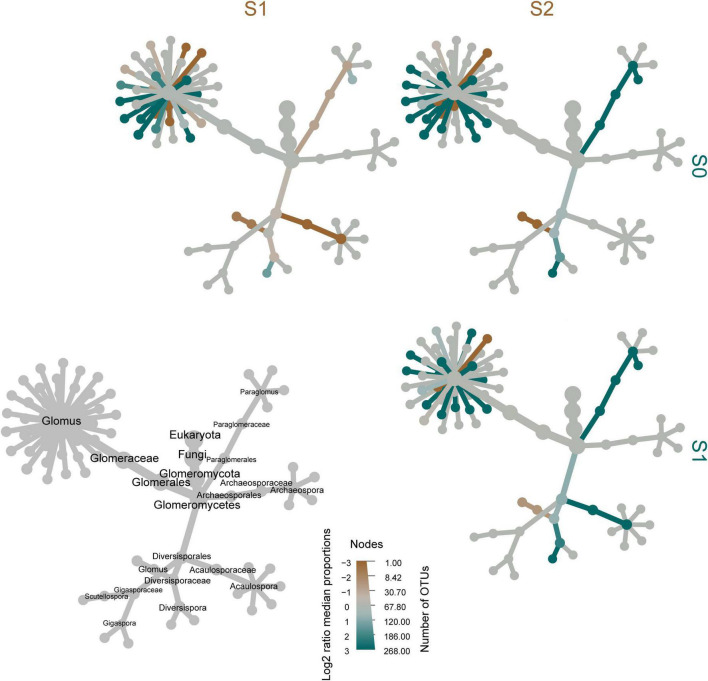
Taxonomic tree for taxa identified in samples. The gray tree on the lower left provides a key for the unlabeled, colored tree matrix. The color of each taxon represents the log2 ratio of median proportions of reads between different sampling sites. Brown taxa are more abundant in the sampling sites depicted in columns and blue taxa are more abundant in the sampling sites in the rows. The size of each node represents the number of operational taxonomic units in the taxon.

A heat tree was used to compare differences in AMF community composition between sampling sites (Figure 3). Several species of *Glomus* were enriched in S0. Paraglomerales and Acaulosporaceae were enriched in S1. With the increase of Sb and As concentrations at the sampling sites, the Diversisporaceae genus *Glomus* was gradually enriched.

### 3.3. Correlation analysis between AMF community composition and soil properties

**FIGURE 4:**
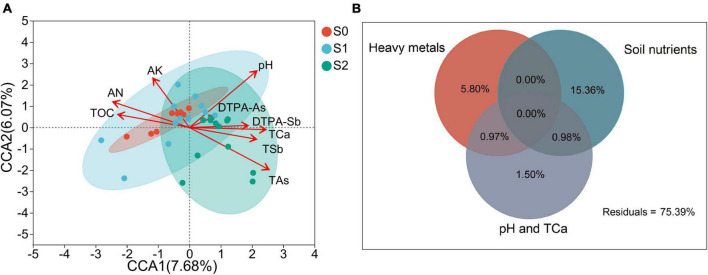
Canonical correspondence analysis (CCA) ordination plots of communities of arbuscular mycorrhizal fungi (AMF) from three sampling sites **(A)** and variation partition analysis (VPA) of the effects of three groups of soil properties on the community composition of arbuscular mycorrhizal fungi **(B)**. The red arrows represent soil properties. The arrow length indicates the degree of influence of soil properties on the AMF community composition. The angles between the arrows of soil properties represent a positive or negative correlation. The circles represent soil samples from different sites. AN, available nitrogen; AK, available potassium; TOC, total organic carbon; TSb, total antimony; DTPA-Sb, diethylenetriamine pentaacetic acid-extractable antimony; TAs, total arsenic; DTPA-As, diethylenetriamine pentaacetic acid-extractable arsenic; TCa, total calcium.

To eliminate multicollinearity, soil properties with VIF > 10 were screened before CCA analysis. The VIF values of the retained soil properties are shown in Supplementary Table 6. CCA was employed to determine the most significant soil properties that shaped the AMF community composition in three sampling sites using retained soil properties (Figure 4A). All of the variables explained 33.75% of the variation in the AMF communities, and only DTPA-As did not affect AMF community composition (Supplementary Table 7). In addition, AN, AK, TOC, pH, TCa, TSb, and TAs extremely significantly affected the community composition of AMF. According to the results of CCA, the soil properties that had a significant impact on the AMF community composition were divided into three groups, namely soil nutrients (including AN, AK, and TOC), heavy metals (including TAs, TSb, and DTPA-Sb), and pH and TCa. Then, VPA was conducted to compare the effects of soil nutrients, heavy metals, and pH and TCa on the AMF community composition (Figure 4B). Totals of 15.36, 5.80, and 1.50% of the variation could be assigned to soil nutrients, heavy metals, and pH and TCa independently.

**FIGURE 5:**
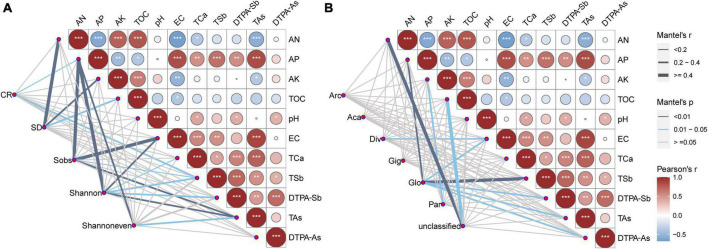
Correlation analysis between soil properties and the AMF colonization rate, spore density, and the Sobs, Shannon, and Shannoneven indexes **(A)**, and between soil properties and AMF families **(B)**. Pairwise comparisons of environmental factors are shown with a color gradient denoting the Pearson correlation coefficients. The edge width corresponds to Mantel’s R statistic for the corresponding distance correlations, and the edge color denotes the statistical significance. AN, available nitrogen; AP, available phosphorus; AK, available potassium; TOC, total organic carbon; TSb, total antimony; DTPA-Sb, diethylenetriamine pentaacetic acid-extractable antimony; TAs, total arsenic; DTPA-As, diethylenetriamine pentaacetic acid-extractable arsenic; EC, electrical conductivity; TCa, total calcium; CR, AMF colonization rate; SD, spore density, Arc, Archaeosporaceae; Aca, Acaulosporaceae; Div, Diversisporaceae; Gig, Gigasporaceae; Glo, Glomeraceae; Par, Paraglomeraceae; unclassified, unclassified Glomeromycetes. *p < 0.05; **p < 0.01; ***p < 0.001.

A Mantel test was used to calculate the correlation between soil properties and the AMF colonization rate, spore density, and the Sobs, Shannon, and Shannoneven indexes (Figure 5A), and between soil properties and AMF families (Figure 5B). TAs was significantly negatively correlated with AN, but positively correlated with AP, EC, TCa, TSb, and DTPA-Sb. TSb was significantly positively correlated with AP, EC, DTPA-Sb, and TAs (Figure 5). The AMF colonization rate correlated with AP (*R* = 0.166, *p* = 0.034) and TSb (*R* = 0.220, *p* = 0.015); spore density was correlated with AN (*R* = 0.401, *p* = 0.003), AK (*R* = 0.337, *p* = 0.004), and TOC (*R* = 0.230, *p* = 0.043); the Sobs index was correlated with AP (*R* = 0.412, *p* = 0.001), EC (*R* = 0.461, *p* = 0.001), TSb (*R* = 0.185, *p* = 0.028), and TAs (*R* = 0.339, *p* = 0.003); the Shannon index was correlated with AP (*R* = 0.462, *p* = 0.001), EC (*R* = 0.377, *p* = 0.003), TCa (*R* = 0.152, *p* = 0.050) TSb (*R* = 0.279, *p* = 0.017), DTPA-Sb (*R* = 0.254, *p* = 0.034), and TAs (*R* = 0.339, *p* = 0.003); and the Shannoneven index was correlated with AP (*R* = 0.318, *p* = 0.007) and TAs (*R* = 0.214, *p* = 0.039) (Figure 5A). Diversisporaceae was correlated with EC (*R* = 0.240, *p* = 0.042); Glomeraceae was correlated with AP (*R* = 0.383, *p* = 0.003), EC (*R* = 0.293, *p* = 0.016), TSb (*R* = 0.416, *p* = 0.007), DTPA-Sb (*R* = 0.321, *p* = 0.034), TAs (*R* = 0.285, *p* = 0.017), and DTPA-As (*R* = 0.257, *p* = 0.038); Paraglomeraceae was correlated with AK (*R* = 0.330, *p* = 0.023); and unclassified Glomeromycetes was correlated with AN (*R* = 0.436, *p* = 0.006) and TOC (*R* = 0.731, *p* = 0.013) (Figure 5B). The Pearson correlation coefficient showed that the AMF colonization rate was positively correlated with AP; spore density was extremely significantly positively correlated with the AN, AK, and TOC and extremely significantly negatively correlated with EC; the Sobs index was positively correlated with AN and negatively correlated with AP, EC, TSb, DTPA-Sb, and TAs; and the Shannon and Shannoneven indexes were negatively correlated with AP, EC, TCa, TSb, DTPA-Sb, and TAs (Supplementary Table 8).

**FIGURE 6:**
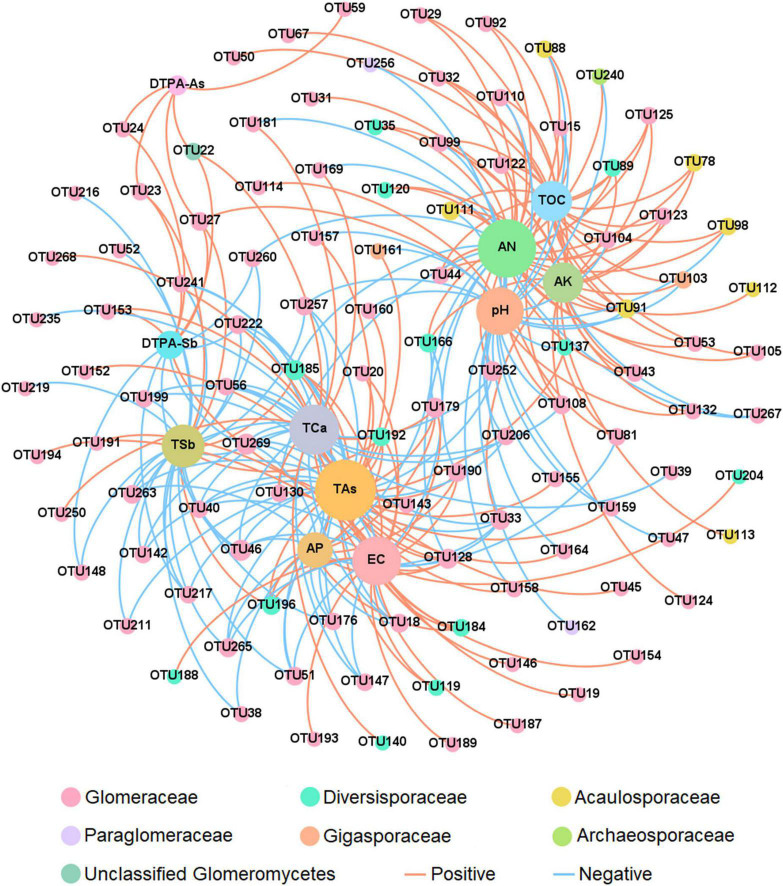
Two-factor network analysis of the association between arbuscular mycorrhizal fungi (AMF) OTUs and environmental factors. The different colors of the species represent the family they belong to. Lines between the points represent a significant correlation (*p* < 0.05) as shown by the Pearson correlation coefficient. Operational taxonomic units (OTUs) were the top 200 species in terms of abundance. AN, available nitrogen; AP, available phosphorus; AK, available potassium; TOC, total organic carbon; TSb, total antimony; DTPA-Sb, diethylenetriamine pentaacetic acid-extractable antimony; TAs, total arsenic; DTPA-As, diethylenetriamine pentaacetic acid-extractable arsenic; EC, electrical conductivity; TCa, total calcium.

Two-factor network analysis result showed that a total of 39 OTUs were correlated with TAs, indicating that TAs was particularly important for the distribution of the AMF community composition, followed by AN (37 OTUs), TCa (30 OTUs), EC (29 OTUs), pH (28 OTUs), TSb (24 OTUs), AK (22 OTUs) and TOC (22 OTUs) (Figure 6). A correlation heatmap was used to demonstrate the correlation between soil properties and AMF richness at the family level (Supplementary Figure 5). The richness of Diversisporaceae was positively correlated with the TSb and TAs. AMF that was unclassified at the family level was positively correlated with DTPA-Sb and DTPA-As.

### 3.4. Integrated responses of the effects of soil properties on AMF richness and diversity

**FIGURE 7:**
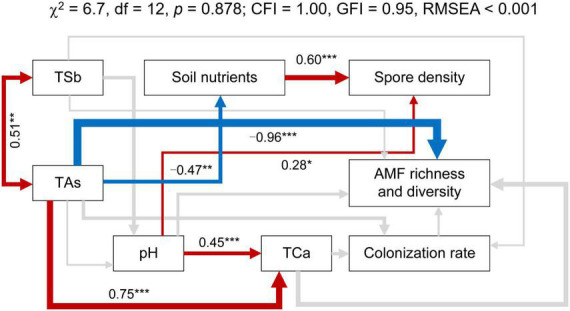
Structural equation model (SEM) showing the hypothesized causal relationships between soil total antimony (TSb), soil total arsenic (TAs), soil total calcium (TCa), pH, soil nutrients, AMF richness and diversity, spore density, and colonization rate. The red and blue arrows indicate positive and negative significant correlations among the indexes, respectively (*p* < 0.05). The arrow width indicates the strength of significant standardized path coefficients. Paths with insignificant coefficients are represented by gray lines. Numbers above the arrows indicate path coefficients. *p < 0.05; **p < 0.01; ***p < 0.001.

The SEM was employed to investigate the integrated responses of the effects of soil properties on AMF richness and diversity (Figure 7). The final model was found to be a good fit to the data (χ^2^ = 6.7, *p* = 0.878; CFI = 1.00, GFI = 0.95, RMSEA < 0.001), accounting for 48, 43, and 13% of the variation in the AMF richness and diversity, the spore density, and the colonization rate, respectively. TAs had a significant direct effect on AMF richness and diversity (λ = −0.96), but the direct effect of TSb on AMF richness and diversity was not significant (λ = −0.21). In addition to the strong direct correlation between TSb and TAs (λ = 0.51), there is also a strong direct correlation between TAs and TCa (λ = 0.77). Moreover, TAs directly affected the content of soil nutrients (λ = −0.53) initially and then indirectly affected AMF spore density (λ = 0.58).

## 4. Discussion

### 4.1. Plant species but not soil properties influenced AMF colonization rate

Arbuscular mycorrhizal fungi (AMF) can colonize the majority of terrestrial plants and form mycorrhizal structures (Kohout et al., 2014; Jiang et al., 2020; Guzman et al., 2021), even in heavy metal-contaminated areas (Sanchez-Castro et al., 2017; Zhan et al., 2019; Riaz et al., 2021). In this study, the roots and rhizosphere soils of five different plants, namely *A. argyi*, *R. acetosa*, *C. abrotanoides*, *B. nivea*, and *B. lindleyana* were collected from areas with different levels of Sb and As contamination, and obvious mycorrhizal infection structures were detected in the roots of these plants (Supplementary Figure 1).

The results showed that the infection structures and colonization rates of these five plants were different (Figure 2A). Multi-way ANOVA analysis showed that the influence of different host plant species on AMF colonization rates was greater than the differences in soil properties caused by spatial heterogeneity (Supplementary Table 3), which was consistent with the results of previous studies (Spruyt et al., 2014; Sanchez-Castro et al., 2017; de Fatima Pedroso et al., 2018). Some studies have suggested that the host plant species is a decisive factor in the colonization of particular AMF species (Teodoro et al., 2020). This may be due to differences in root structure or plant nutrient requirements (Karanika et al., 2008). It was interesting to find that Sb and As contamination did not significantly affect the AMF colonization rates (Figure 5A), which was different from the results of Yang et al. (2016). However, a large number of previous studies were consistent with the results of the present study (Ruotsalainen et al., 2007; Schneider et al., 2016). Teodoro et al. (2020) indicated that differences in the frequency and intensity of colonization were found for different plant species, but that the concentrations of Zn, Pb, As, and Cd in rhizospheres and plants were poorly correlated with AMF colonization. Malcová et al. (2003) conducted a cultivation experiment and found that the addition of Pb at different concentrations did not change the percentage of root colonization.

### 4.2. Spore density was not directly affected by soil Sb and As content

The AMF spore density varied greatly in different sampling sites (Figure 2B). Unlike the colonization rates, the AMF spore density was mainly affected by soil properties rather than the host plants (Supplementary Table 3), which was consistent with previous studies (Birhane et al., 2017; Coutinho et al., 2019; Guo et al., 2020). However, the results showed that Sb and As content in soil was still not the main factor affecting the AMF spore density. At present, there are different opinions about the effect of heavy metals on AMF spore density, including a positive correlation (Krishnamoorthy et al., 2015), a negative correlation (Zarei et al., 2008, 2010; Schneider et al., 2013), and no correlation (Yang et al., 2015). This phenomenon may be caused by the different sensitivities of different AMFs to different heavy metals (Pawlowska and Charvat, 2004). In addition, the effects of heavy metals on the sporulation process of AMF may depend on other environmental factors (Yang et al., 2015). In this study, it was found that the AMF spore density was positively correlated with AN, and TOC (Figures 5A, 7), which is consistent with the results of previous studies (Bhat et al., 2014; de Araujo Pereira et al., 2018; Amir et al., 2022). It is generally believed that the AMF spore density is positively correlated with soil nitrogen and organic matter when the soil nutrient content is low (Moradi et al., 2017). For example, Zhang et al. (2021) indicated that increasing the C:N ratio favored sporulation. Another reason for this phenomenon may be related to the glomalin secreted by AMF spore, which is one of the major sources of soil organic carbon and nitrogen (Guo et al., 2020). However, TAs most likely affects soil spore density by affecting soil N, TOC, and other nutrients. In the present study, TAs was found to be negatively correlated with soil AN, AK, and TOC (Figures 5, 7). Previous studies have shown that As contamination can significantly reduce soil microbial biomass nitrogen and microbial biomass carbon, which are important sources of soil nitrogen and organic carbon (Ghosh et al., 2004). In addition, a large number of studies have confirmed that soil As pollution reduces soil biodiversity (Yu et al., 2021; Jia et al., 2022) and then affects the cycling of C, N, and other elements in the soil.

### 4.3. As had a stronger effect on AMF community composition than Sb

It was found that the AMF community composition was significantly affected by different plant species and sampling sites (Supplementary Figure 3 and Supplementary Table 5). There was no significant difference in AMF community composition between *A. argyi* and *R. acetosa* rhizosphere soils, the differences were mainly in the rhizosphere soils of *C. abrotanoides*, *B. nivea*, and *B. lindleyana*. This difference may have been because these plants were located at different sampling sites, and the differences in the AMF community composition between sampling sites led to the differences in the AMF community composition in these plant rhizosphere soils. In this study, the AMF community composition was different among sampling sites (Supplementary Figure 3 and Supplementary Table 5). Because all of the sampling sites were located in Qinglong County, Qianxinan Buyei and Miao Autonomous Prefecture, Guizhou Province, the differences in climate between the sampling sites could be ignored. Therefore, such differences in AMF community composition were more likely to be related to differences in soil properties. Many studies have demonstrated that soil properties can significantly affect the AMF community composition (Wu et al., 2020; Silva et al., 2022). The Sobs, Shannon, Shannoneven, and Coverage indexes were used to evaluate the AMF richness, diversity, evenness, and coverage at different sampling sites, respectively (Figures 2C–F). The results showed that the order of AMF richness and diversity of the three sampling sites was S0 > S1 > S2. Multi-way ANOVA analysis showed that the variation of soil properties was the dominant factor influencing AMF diversity (Supplementary Table 3), which was consistent with the results on spore density (Supplementary Table 3) and AMF community composition (Supplementary Table 5). Previous studies have also supported these phenomena (Faggioli et al., 2019; Faghihinia et al., 2021).

To further understand which soil properties were the main influencing factors for AMF community composition, CCA analysis was performed after multivariate collinear screening (Figure 4 and Supplementary Table 6). CCA analysis showed that eight soil properties had significant effects on the AMF community composition, namely AN, AK, TOC, pH, TCa, TSb, DTPA-Sb, and TAs. It should be noted that AP and EC were removed due to multicollinearity with other environmental factors (Supplementary Table 6). The Pearson correlation coefficient showed that there was an extremely strong correlation between AP, EC, and TAs (Pearson’s *R* > 0.8) (Supplementary Table 9), and the effect of AP and EC on the AMF community composition could be approximated by TAs. All the soil properties that significantly affected AMF community composition were divided into three groups according to their attributes, namely soil nutrients, heavy metals, and pH and TCa. VPA analysis was used to compare the effects of these three groups of soil properties on the AMF community composition (Figure 4B). The results showed that soil nutrients were the most important factors affecting the AMF community composition. The effects of soil nutrient content (Yan K. et al., 2021; Yan T. et al., 2021; Emery et al., 2022), pH (Van Geel et al., 2018; Xiao et al., 2022), and heavy metal content (Zarei et al., 2010; Faggioli et al., 2019) on the AMF community composition have been extensively demonstrated. However, few studies have discussed which group of soil properties has a greater impact on AMF community composition. Stefanowicz et al. (2020) indicated that although heavy metal pollution was a meaningful agent shaping soil microbial communities, the toxic effect of heavy metals was weaker than the organic matter content, which was consistent with the present results. In contrast, Zhang et al. (2019) found that the soil pH and EC were more important than the available N, P, and K drivers of the variation of AMF diversity in saline-sodic soils, which was different from the results of this study. This may be due to the complex interrelationship between soil properties, groups, models, and soil types will affect the analysis results. Understanding the dominant factors that induce changes in the AMF community composition is of great practical significance for the design of AMF application techniques.

A Mantel test was used to investigate the effects of soil properties on the richness, diversity, and evenness of AMF, AP, EC, TCa, TSb, DTPA-Sb, and TAs, which significantly affected the AMF richness and diversity (Figure 5). Thirty-nine and 24 OTUs were closely related to TAs and TSb (Figure 6), respectively, thus indicating that both TSb and TAs greatly affected the AMF community composition, but TAs had a greater effect than TSb. As expected, both TAs and TSb were significantly negatively correlated with AMF richness and diversity (Supplementary Table 8), which confirmed the majority view that heavy metal pollution would reduce AMF richness and diversity (Faggioli et al., 2019; Parvin et al., 2019). Excessive heavy metal concentrations are toxic to most AMFs. Only AMFs that exhibit strong heavy metal tolerances can survive, which reduces AMF diversity (Pawlowska and Charvat, 2004; Faggioli et al., 2019). Although Schneider et al. (2013) concluded that As contamination reduced the AMF species richness in an investigation of the AMF community composition in a gold mining area in Minas Gerais State, Brazil, they did not systematically analyze the correlation between soil As content and other properties and AMF diversity. The results of the present study provide direct evidence for this conclusion. Wei et al. (2015a) also investigated the AMF community composition in an Sb mining area and preliminarily concluded that Sb dominated the distribution of the AMF community composition. However, their study only considered the effects of Sb, P, N, pH, and organic matter on the AMF community composition, and it did not take the As content into account. Sb and As often co-occur in landscapes impacted by mining activities (Johnston et al., 2020); therefore, the influence of As pollution in Sb mining areas cannot be ignored. The present study provides direct evidence that although Sb has a significant effect on the AMF community composition, As is the more important factor affecting the AMF community composition in Sb and As mining areas.

It is worth noting that there was a strong positive correlation between AP, TAs, and EC (Figure 5 and Supplementary Table 9). AP has been widely confirmed to be an important factor affecting AMF community composition (Zarei et al., 2010). There is competitive adsorption between As and P in soil (Gunes et al., 2008). P can be desorbed by As due to a mass action effect of high As concentrations (Anawar et al., 2018), and it can then improve the availability of P. Numerous studies have shown that interactions between As and P in the soil can significantly affect the availability of As and P (Wu et al., 2022). There are reciprocal reward mechanisms between AMF and host plants (Kiers et al., 2011), wherein AMF can provide mineral nutrients to host plants (especially P) and other benefits, including protection against biotic and abiotic stresses (Smith et al., 2009; Huang et al., 2019). In exchange, plants supply AMF with carbohydrates, which are essential and necessary for fungal survival and growth (Parniske, 2008). High soil P content allows plants to take up P without resorting to mycorrhizal symbionts, which in turn reduces the supply of carbohydrates from the host plants to the symbiotic AMF (Johnson et al., 2013). Therefore, this study indicated that the effect of soil As on AMF community composition cannot be simply attributed to the toxic effect of As on AMF, but should be caused by the joint effect of As and P.

In addition, the present study found that AMF richness was positively correlated with AN, while AMF richness and diversity were negatively correlated with TCa (Supplementary Table 8). Some genera of AMF need to obtain more organic acids from microbial metabolism when microbial biomass increases, thus leading to a significant correlation between the AMF richness and microbial biomass N (Xiao D. et al., 2019). Xiao et al. (2022) found that the decreases in microbial motility caused by high Ca resulted in the decline of some AMF groups and the selection of certain AMF species (Tang et al., 2019), leading to a significantly negative correlation between the AMF diversity and Ca content.

Glomeraceae was detected as the dominant family in all of the groups of samples in this study (Supplementary Figure 2). Numerous studies have suggested that Glomeraceae exhibits strong ecological adaptability and it is the dominant family in areas under heavy metal stress (Zarei et al., 2008; Long et al., 2010; Yang et al., 2015; Sanchez-Castro et al., 2017; Faggioli et al., 2019), including areas contaminated with Sb (Wei et al., 2015a) or As (Parvin et al., 2019). One possible reason is that Glomeraceae can repair damaged hyphae in stressful environments, which helps to improve the ability of these fungi to adapt to environmental stresses (de la Providencia et al., 2005; Huang et al., 2019). Another reason is that plants harbor Glomeraceae that can benefit their growth and improve their competitiveness under specific environmental conditions (Wei et al., 2015b; Huang et al., 2019), thereby promoting the dominance of Glomeraceae in soil ecosystems. Other studies suggest that the predominant detection of Glomeraceae might be explained by their high sporulation rate (Sanchez-Castro et al., 2017). Previous studies have demonstrated that Glomeraceae can form beneficial symbiotic relationships with various plants, and enhance the efficiency of the ecological restoration of heavy metal-contaminated soil by promoting the growth or heavy metal absorption of plants (Riaz et al., 2021). Glomeraceae is widely distributed, even in Sb- and As-polluted soil, and it seems to be a potential option to promote ecological remediation efficacy in Sb- and As-contaminated areas. In addition, the present study found that TSb and TAs significantly affected the community structure of the Glomeraceae (Figures 3, 5B, 6). This phenomenon indicates that even AMFs of the same family have different sensitivities to heavy metals (Faggioli et al., 2019). Interestingly, the study found that the richness of Diversisporaceae was positively correlated with the TSb and TAs (Figure 3 and Supplementary Figure 5). Parvin et al. (2019) found three AMF families that predominated in most As-contaminated soils, namely Glomeraceae, Claroideoglomeraceae, and Diversisporaceae. The results of this study supported this research phenomenon and directly confirmed that there was a positive linear correlation between Diversisporaceae and soil TSb and TAs. This finding may be due to the high Sb and As tolerance of Diversisporaceae itself, leading to its high relative abundance in soil with high concentrations of Sb and As.

The SEM demonstrated the relationship between soil environmental factors and the AMF richness and diversity, spore density, and the colonization rate in the system examined in this study (Figure 7). It has been well documented that Sb and As often co-exist and co-contaminate adjacent regions (Okkenhaug et al., 2012; Li Y. et al., 2021). Therefore, TSb and TAs are positively correlated, and together they negatively affect AMF richness and diversity, with TAs having an extremely significant effect on AMF richness and diversity. There was a negative correlation between soil nutrients (mainly AN, AK, and TOC) and TAs. High levels of As contamination decrease soil nutrition cycling by inhibiting the growth of soil animals, plants, and microbes (Jiao et al., 2016). In addition, Yu et al. (2021) found that high Sb and As stress inhibited the microbial carbon mineralization and nitrification processes in the plant rhizosphere soils and further reduced soil enzymatic activities, carbon mineralization, and nitrification potential, of which the disruption caused by As was more obvious. The positive relationship between soil nutrients and spore density was mainly because soil C and N promoted AMF sporulation (Zhang et al., 2021).

## 5. Conclusion

The present study identified the AMF community composition and its main influencing factors in Sb- and As-contaminated areas. Glomeraceae was the dominant family in Sb and As mining areas. In Sb- and As-polluted areas, the affinity between AMF and plants is mainly affected by plant species, and less affected by soil Sb and As content. Spore density is mainly affected by soil nutrients, such as AN, AK, and TOC, but soil TAs contamination may be important environmental property sleading to low soil nutrient content. The effect of soil nutrients on AMF community composition was also greater than that of TSb and TAs contamination. However, soil contamination with Sb and As also had a significant effect on AMF community composition, with the effect of TAs being significantly greater than that of TSb. The effect of TAs on AMF community composition cannot be attributed to the toxic effect of As on AMF alone, and the interaction of As and P is also an important reason for the reduction of soil AMF richness and diversity. Therefore, the potential impact of As on the effective application of mycorrhizal technology should be further considered when this technology is applied to the ecological restoration of Sb- and As-contaminated areas.

## Data availability statement

The datasets presented in this study can be found in online repositories. The names of the repository/repositories and accession number(s) can be found in the article/Supplementary material.

## Author contributions

YM: investigation, data curation, methodology, software, visualization, and writing—original draft preparation. XRL, MZ, KC, CD, and XML: investigation. CX, NJ, FW, HS, and XSL: manuscript revision. YW: conceptualization, data curation, manuscript revision, and funding acquisition. All authors contributed to the article and approved the submitted version.

## Footnotes

1
http://drive5.com/uparse/

2
http://rdp.cme.msu.edu/

3
https://mothur.org/wiki/calculators/
