# Supplementary figures and images for: Arbuscular mycorrhizal fungi community analysis revealed the significant impact of arsenic in antimony- and arsenic-contaminated soil in three Guizhou regions
Source: Front Microbiol. 2023 May 18;14:1189400. doi: 10.3389/fmicb.2023.1189400 (PMC10232906; doi:10.3389/fmicb.2023.1189400)

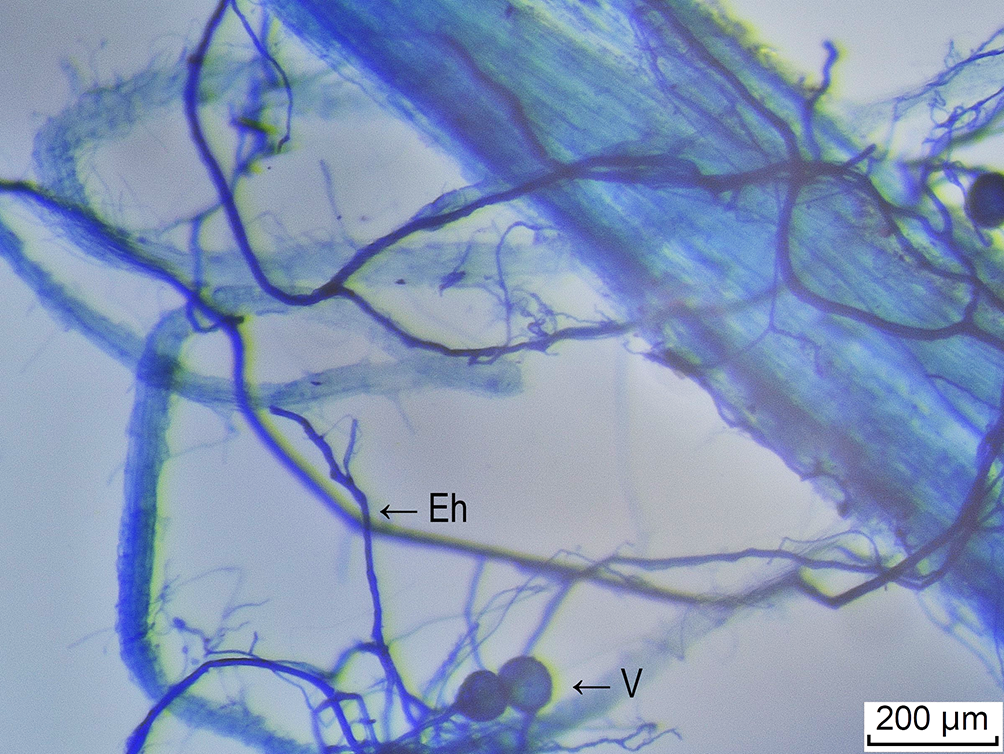

Supplement: Supplementary file 1 [file Image_1.TIF]

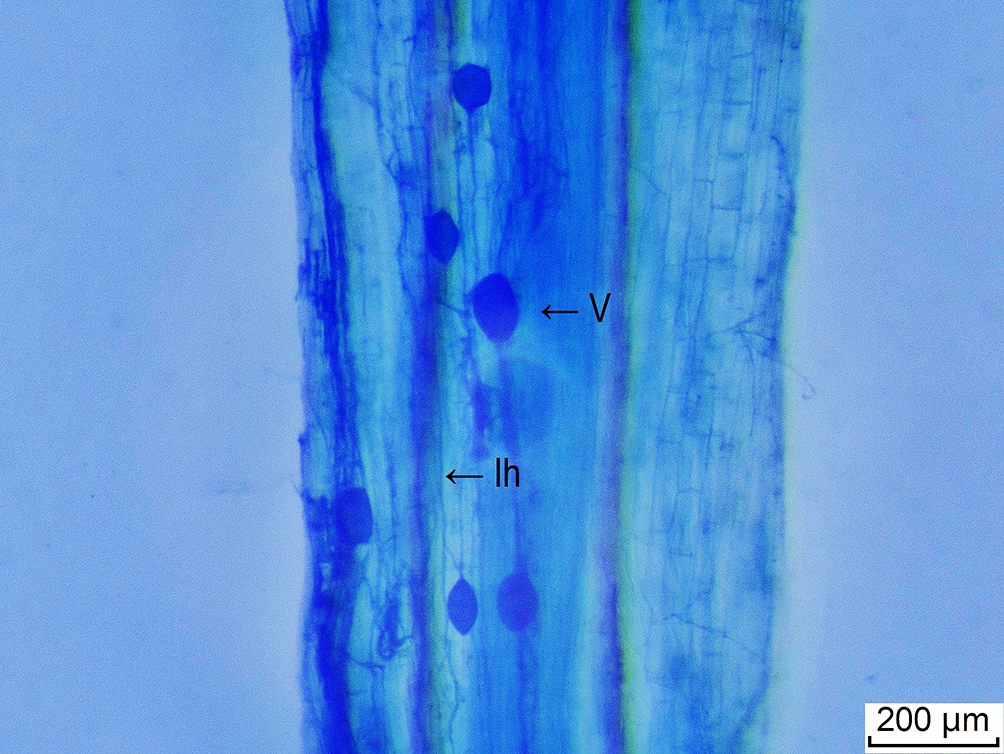

Supplement: Supplementary file 2 [file Image_2.TIF]

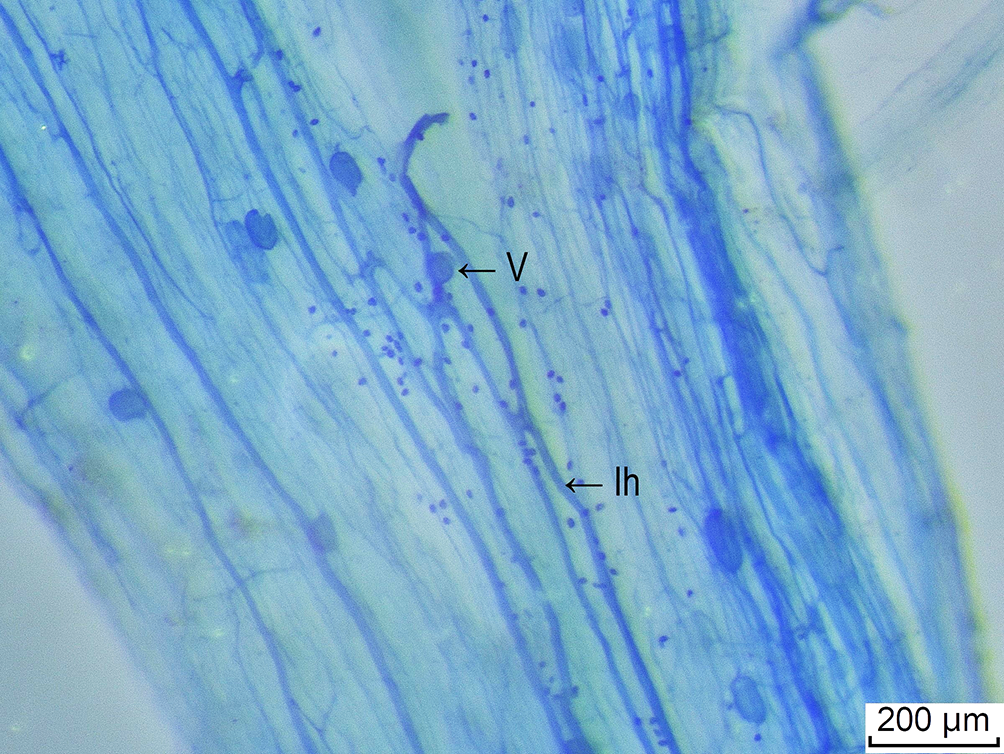

Supplement: Supplementary file 3 [file Image_3.TIF]

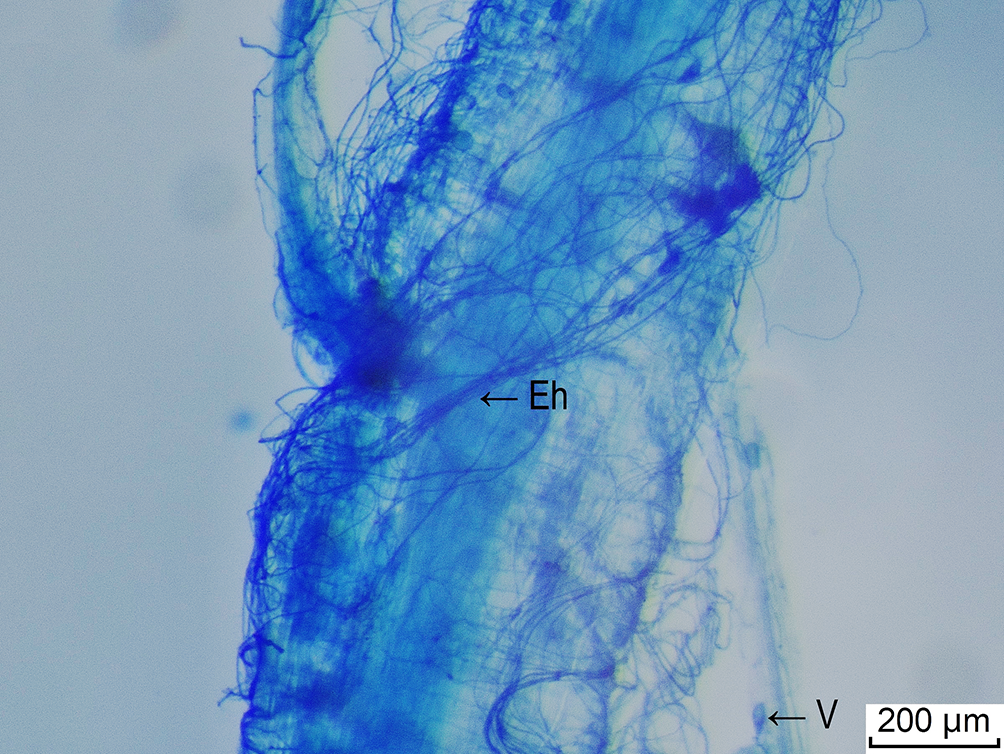

Supplement: Supplementary file 4 [file Image_4.TIF]

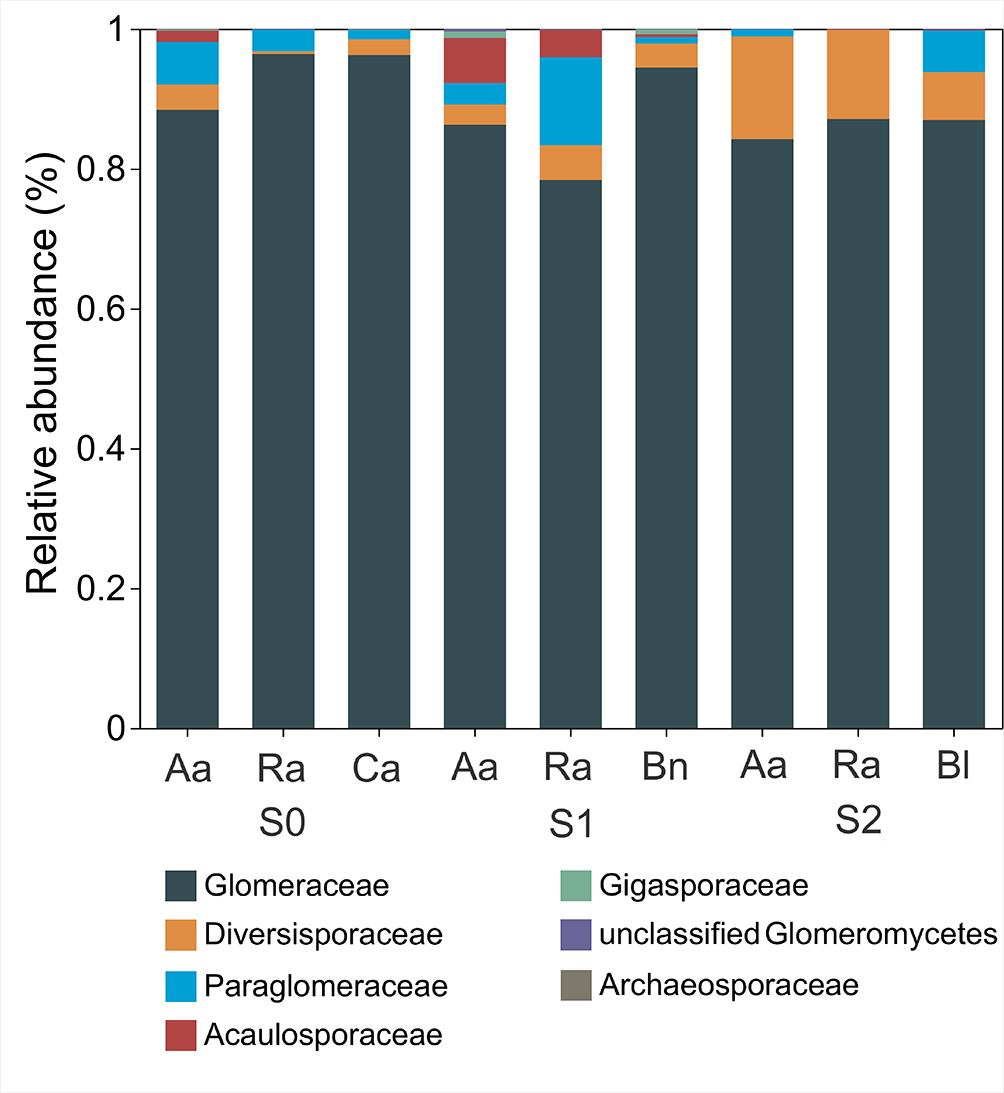

Supplement: Supplementary file 5 [file Image_5.TIFF]

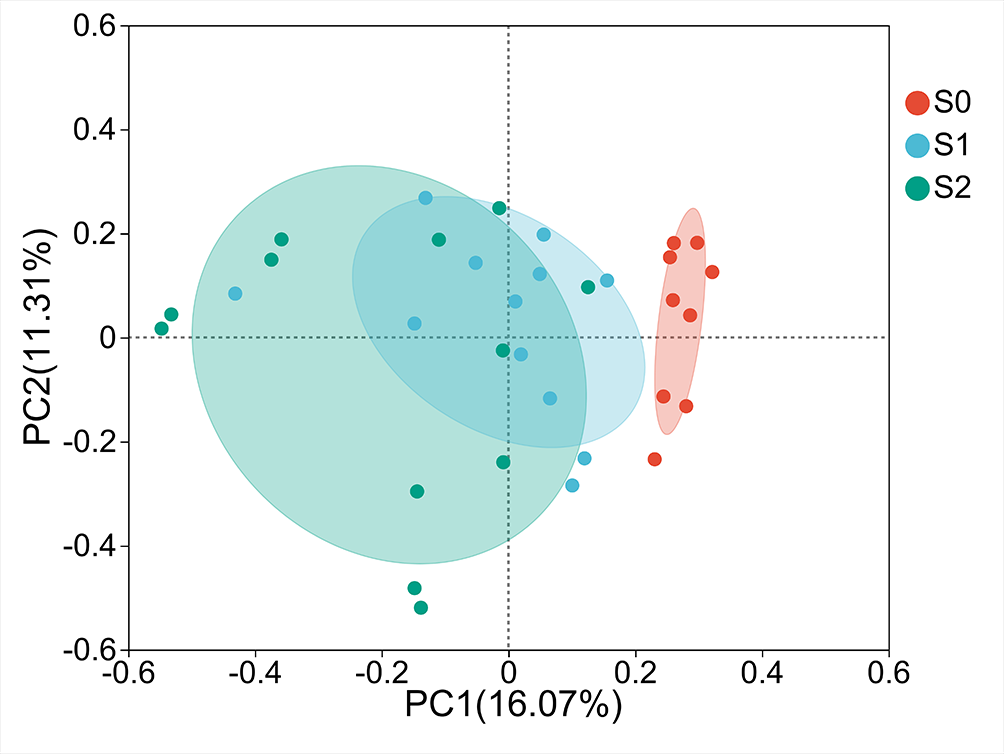

Supplement: Supplementary file 6 [file Image_6.TIFF]

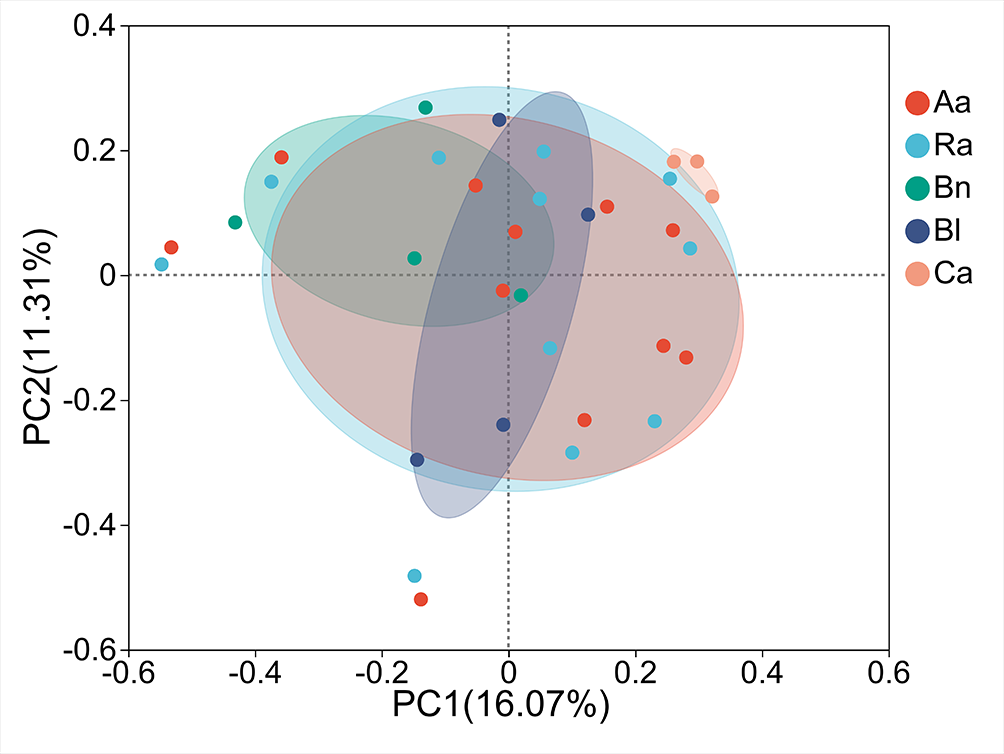

Supplement: Supplementary file 7 [file Image_7.TIFF]

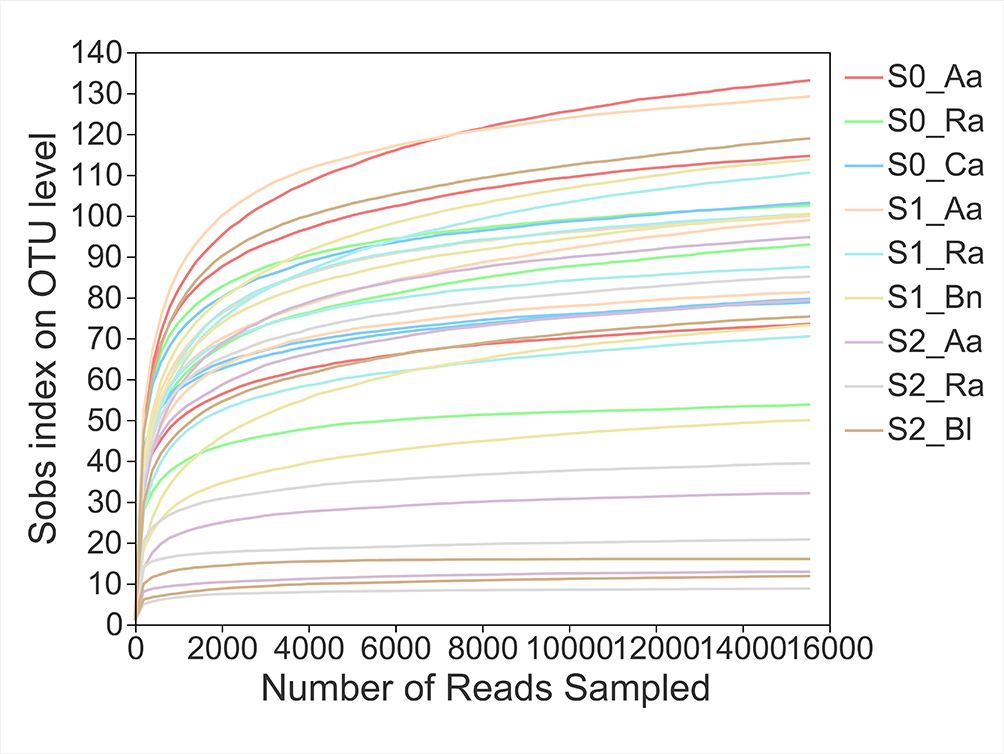

Supplement: Supplementary file 8 [file Image_8.TIFF]

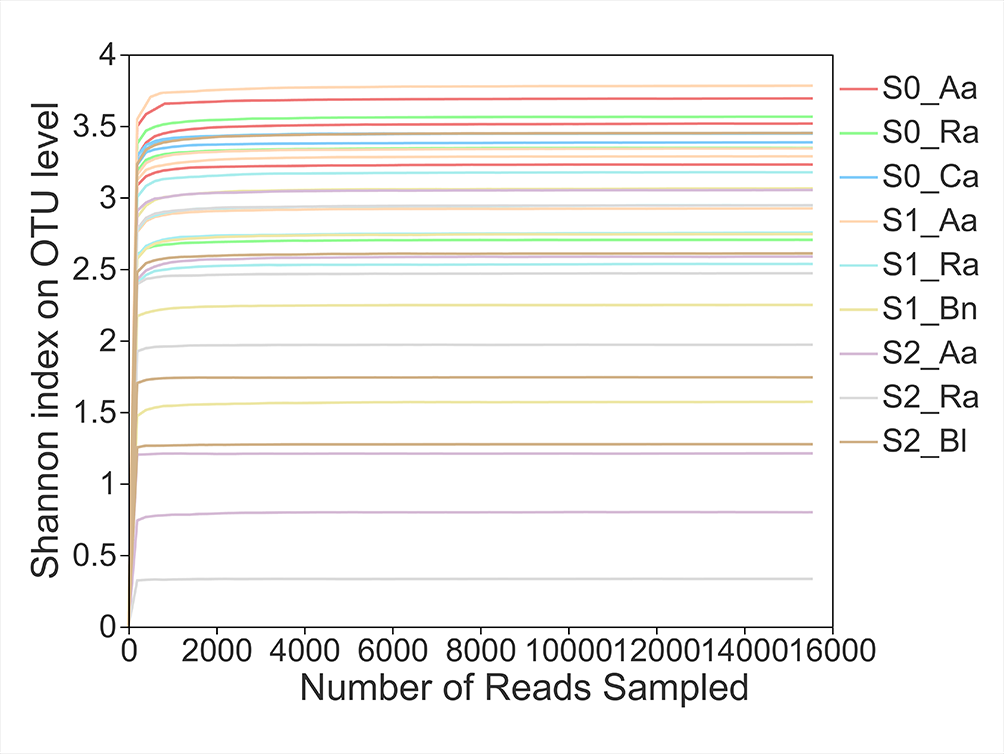

Supplement: Supplementary file 9 [file Image_9.TIFF]

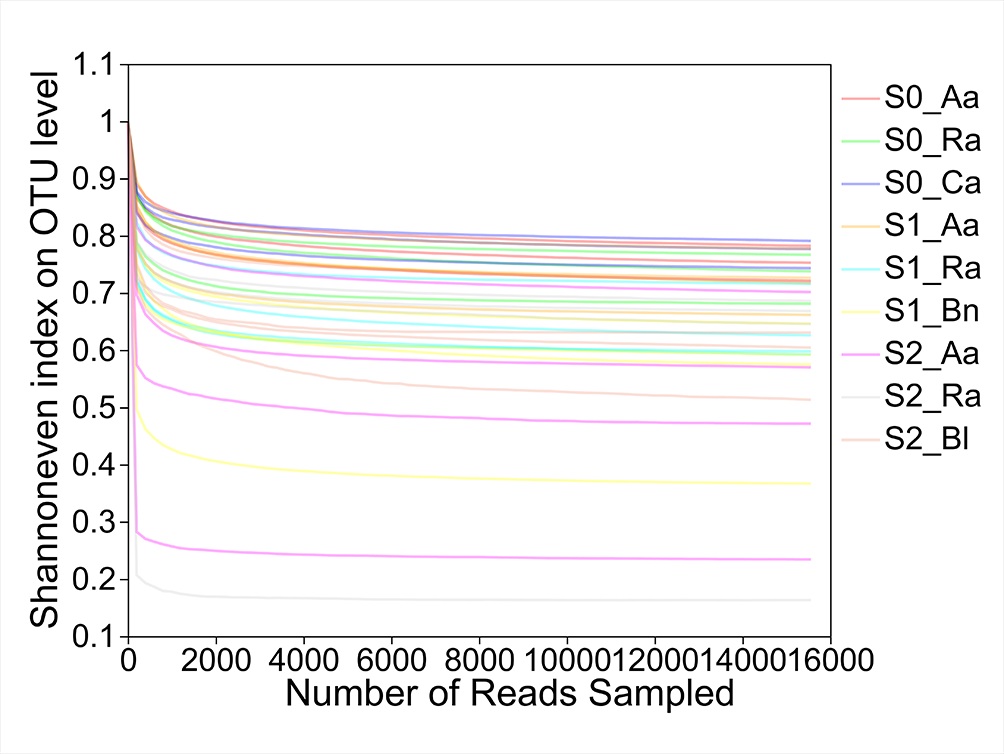

Supplement: Supplementary file 10 [file Image_10.TIFF]

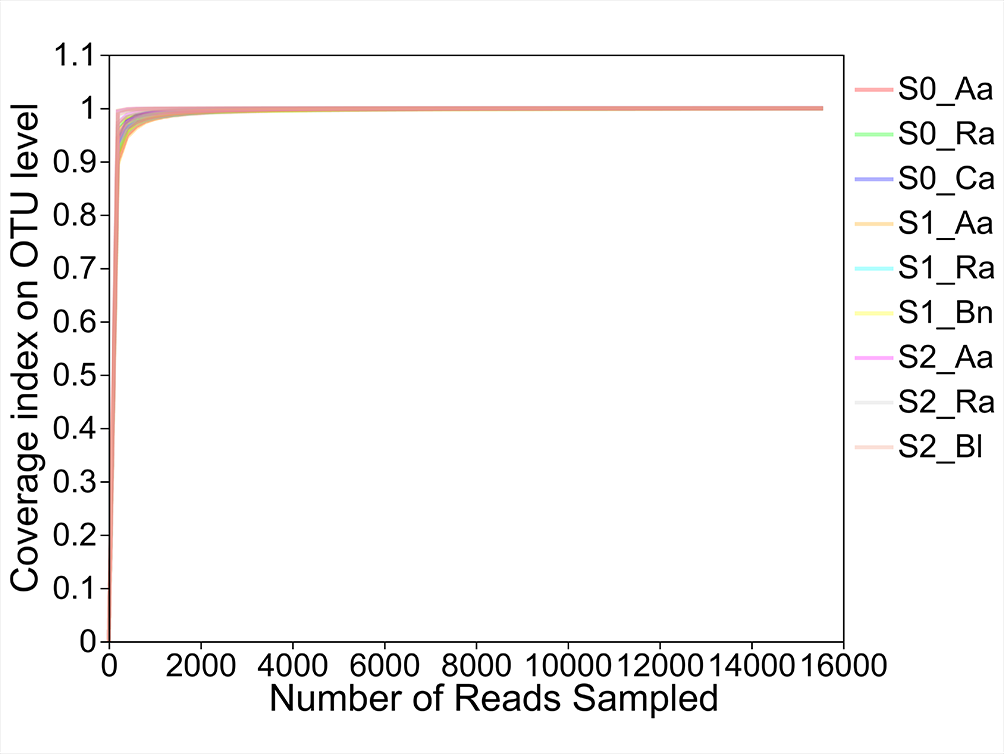

Supplement: Supplementary file 11 [file Image_11.TIFF]

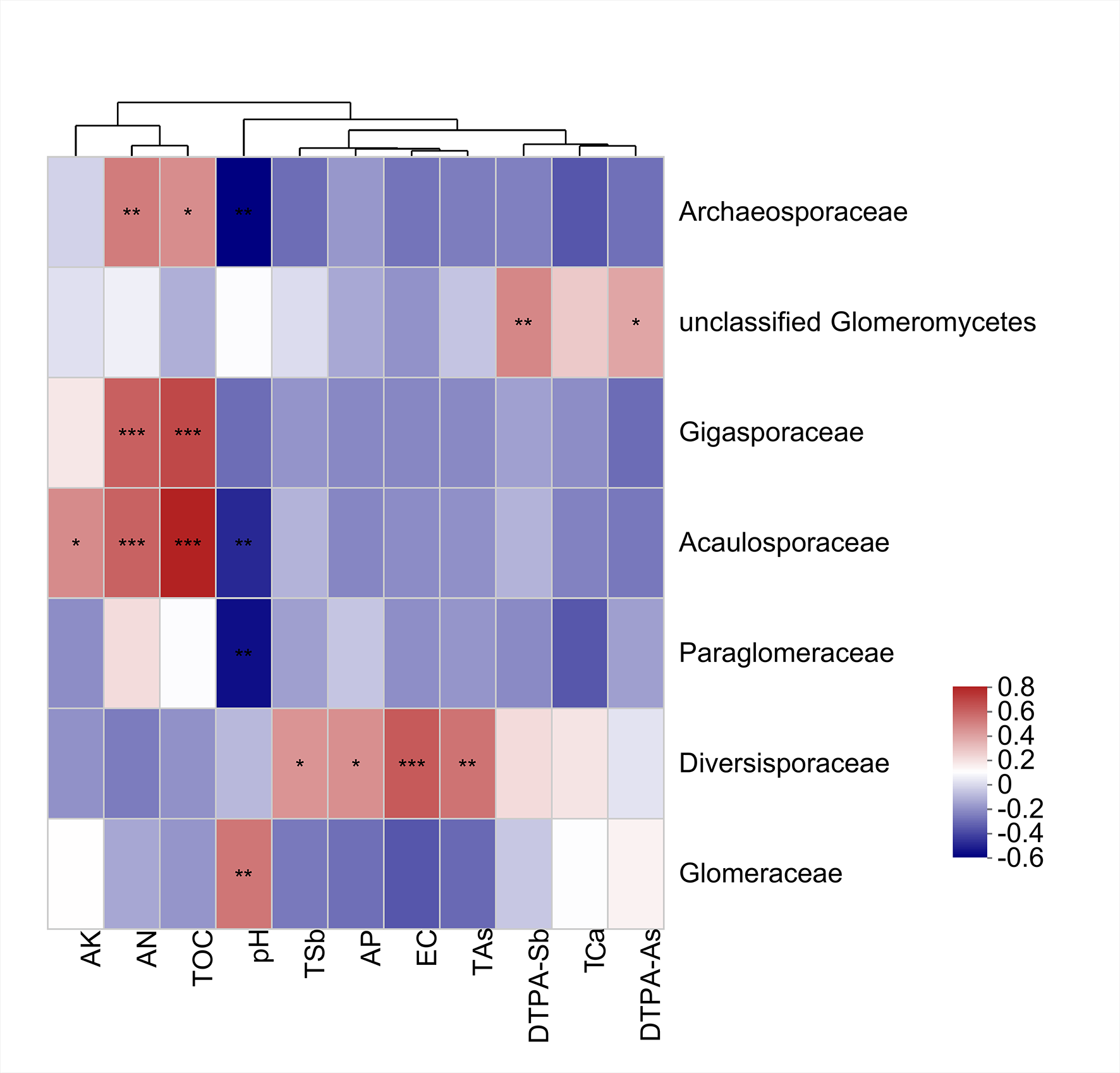

Supplement: Supplementary file 12 [file Image_12.TIFF]
