# Supplementary material for: Arbuscular mycorrhizal fungi community analysis revealed the significant impact of arsenic in antimony- and arsenic-contaminated soil in three Guizhou regions
Source: Front Microbiol. 2023 May 18;14:1189400. doi: 10.3389/fmicb.2023.1189400 (PMC10232906; doi:10.3389/fmicb.2023.1189400)
Supplement: Supplementary file 13 [file Table_1.docx]

**Supplementary Table 1.** Location information for sampling sites.

| Sampling site | Latitude and longitude | Elevation (m) | Area (m^2^) | Dominant plants |
| --- | --- | --- | --- | --- |
| S0 | 105°9′17.68″–105°9′25.11″ E  25°41′4.63″–25°41′8.40″ N | 1831.3 | 4717.2 | *Artemisia argyi*  *Rumex acetosa*  *Boehmeria nivea* |
| S1 | 105°9′10.26″–105°9′15.95″ E,  25°40′48.51″–25°40′51.93″ N | 1805.2 | 10603.3 | *Artemisia argyi*  *Rumex acetosa*  *Buddleja lindleyana* |
| S2 | 105°10′36.35″–105°10′41.78″ E  25°40′12.03″–25°40′17.21″ N | 1586.9 | 12031.6 | *Artemisia argyi*  *Rumex acetosa*  *Carpesium abrotanoides* |
