# Supplementary material for: Arbuscular mycorrhizal fungi community analysis revealed the significant impact of arsenic in antimony- and arsenic-contaminated soil in three Guizhou regions
Source: Front Microbiol. 2023 May 18;14:1189400. doi: 10.3389/fmicb.2023.1189400 (PMC10232906; doi:10.3389/fmicb.2023.1189400)
Supplement: Supplementary file 14 [file Table_2.docx]

**Supplementary Table 2.** Polymerase chain reaction (PCR) program and reaction system components.

| First round | | | |
| --- | --- | --- | --- |
| Reaction components | | Volume/Mass | |
| 5 × FastPfu Buffer | | 4 μL | |
| 2.5 mM deoxy-ribonucleoside triphosphates (dNTPs) | | 2 μL | |
| AML1 | | 0.8 μL | |
| AML2 | | 0.8 μL | |
| FastPfu polymerase | | 0.4 μL | |
| Template DNA | | 10 ng | |
| ddH_2_O | | 20 μL | |
| Reaction parameters | | Temperature (°C) | Time (s) |
| Initial denaturation | | 95 | 180 |
| 25 cycles | Denaturation | 95 | 30 |
|  | Annealing | 55 | 30 |
|  | Extension | 72 | 45 |
| Final extension | | 72 | 600 |
| Refrigeration | | 10 |  |
| Second round | | | |
| Reaction components | | Volume/Mass | |
| 5 × FastPfu Buffer | | 4 μL | |
| 2.5 mM deoxy-ribonucleoside triphosphates (dNTPs) | | 2 μL | |
| AMV4.5NF | | 0.8 μL | |
| AMDGR | | 0.8 μL | |
| FastPfu polymerase | | 0.4 μL | |
| Template DNA | | 10 ng | |
| ddH_2_O | | 20 μL | |
| Reaction parameters | | Temperature (°C) | Time (s) |
| Initial denaturation | | 95 | 180 |
| 30 cycles | Denaturation | 95 | 30 |
|  | Annealing | 55 | 30 |
|  | Extension | 72 | 45 |
| Final extension | | 72 | 600 |
| Refrigeration | | 10 |  |
