# Supplementary material for: Arbuscular mycorrhizal fungi community analysis revealed the significant impact of arsenic in antimony- and arsenic-contaminated soil in three Guizhou regions
Source: Front Microbiol. 2023 May 18;14:1189400. doi: 10.3389/fmicb.2023.1189400 (PMC10232906; doi:10.3389/fmicb.2023.1189400)
Supplement: Supplementary file 15 [file Table_3.docx]

**Supplementary Table 3.** Significance levels (F values) of the effects of plants, sampling sites, and their interactions on the arbuscular mycorrhizal fungi (AMF) colonization rate, the spore density, and the Sobs, Shannon, Shannoneven, and Coverage indexes according to a multi-way analysis. of variance (ANOVA).

| Index | Group | SS | Df | MS | F value | *p* value | Partial eta square |
| --- | --- | --- | --- | --- | --- | --- | --- |
| AMF colonization rate | Plant | 0.263 | 4 | 0.066 | **3.488** | **0.022** | 0.368 |
|  | Sampling sites | 0.049 | 2 | 0.025 | 1.301 | 0.291 | 0.098 |
|  | Plant × Sampling sites | 0.050 | 2 | 0.025 | 1.325 | 0.284 | 0.099 |
| Spore density | Plant | 1872.667 | 4 | 468.167 | 0.982 | 0.436 | 0.141 |
|  | Sampling sites | 32407.530 | 2 | 16203.765 | **33.978** | **0.001** | 0.739 |
|  | Plant × Sampling sites | 928.273 | 2 | 464.136 | 0.973 | 0.392 | 0.075 |
| Sobs index on OTU level | Plant | 2603.322 | 4 | 650.831 | 0.593 | 0.671 | 0.090 |
|  | Sampling sites | 13255.068 | 2 | 6627.534 | **6.041** | **0.007** | 0.335 |
|  | Plant × Sampling sites | 129.341 | 2 | 64.670 | 0.059 | 0.943 | 0.005 |
| Shannon index on OTU level | Plant | 1.921 | 4 | 0.480 | 0.921 | 0.468 | 0.133 |
|  | Sampling sites | 8.547 | 2 | 4.274 | **8.198** | **0.002** | 0.406 |
|  | Plant × Sampling sites | 0.251 | 2 | 0.125 | 0.241 | 0.788 | 0.020 |
| Shannoneven  index on OTU level | Plant | 0.076 | 4 | 0.019 | 1.153 | 0.356 | 0.161 |
|  | Sampling sites | 0.197 | 2 | 0.098 | **5.987** | **0.008** | 0.333 |
|  | Plant × Sampling sites | 0.016 | 2 | 0.008 | 0.492 | 0.617 | 0.039 |
| Coverage index on OTU level | Plant | <0.001 | 4 | <0.001 | 0.214 | 0.928 | 0.034 |
|  | Sampling sites | <0.001 | 2 | <0.001 | 2.618 | 0.094 | 0.179 |
|  | Plant × Sampling sites | <0.001 | 2 | <0.001 | 0.618 | 0.547 | 0.049 |

Note: SS: Sum of squares; Df: Degree of freedom; MS: Mean square; OTU: operational taxonomic unit.
