# Supplementary material for: Arbuscular mycorrhizal fungi community analysis revealed the significant impact of arsenic in antimony- and arsenic-contaminated soil in three Guizhou regions
Source: Front Microbiol. 2023 May 18;14:1189400. doi: 10.3389/fmicb.2023.1189400 (PMC10232906; doi:10.3389/fmicb.2023.1189400)
Supplement: Supplementary file 16 [file Table_4.docx]

**Supplementary Table 4.** Operational taxonomic units (OTUs) and sequence numbers of related species for each sampling site.

| Family | Genus | Species | OTU | S0-Aa1 | S0-Ra1 | S0-Ca1 | S0-Aa2 | S0-Ra2 | S0-Ca2 | S0-Aa3 | S0-Ra3 | S0-Ca3 | S1-Aa1 | S1-Ra1 |
| --- | --- | --- | --- | --- | --- | --- | --- | --- | --- | --- | --- | --- | --- | --- |
| Archaeosporaceae | *Archaeospora* | *Archaeospora-Aca*-VTX00338 | 3 | 0 | 0 | 0 | 0 | 0 | 0 | 0 | 0 | 0 | 0 | 1 |
|  |  | *Archaeospora-Other1*-VTX00005 | 2 | 0 | 0 | 0 | 0 | 0 | 0 | 34 | 3 | 0 | 0 | 0 |
|  |  | *Archaeospora-sp.*-VTX00009 | 2 | 1 | 0 | 0 | 0 | 0 | 0 | 0 | 1 | 0 | 0 | 0 |
|  |  | *Archaeospora-trappei*-VTX00245 | 1 | 0 | 0 | 0 | 0 | 0 | 0 | 0 | 0 | 0 | 0 | 0 |
|  |  | Unclassified *Archaeospora* | 2 | 10 | 0 | 0 | 0 | 0 | 0 | 0 | 0 | 0 | 0 | 1 |
|  | norank | *unknown-Wirsel-OUT21*-VTX00004 | 1 | 0 | 0 | 0 | 0 | 0 | 0 | 0 | 0 | 0 | 0 | 0 |
| Acaulosporaceae | *Acaulospora* | *Acaulospora-Acau11*-VTX00031 | 1 | 0 | 0 | 0 | 0 | 0 | 0 | 0 | 0 | 0 | 0 | 0 |
|  |  | *Acaulospora-Acau12*-VTX00045 | 1 | 25 | 0 | 0 | 0 | 0 | 0 | 51 | 0 | 0 | 0 | 0 |
|  |  | *Acaulospora-Acau8*-VTX00027 | 1 | 0 | 0 | 0 | 0 | 0 | 0 | 0 | 0 | 0 | 0 | 0 |
|  |  | *Acaulospora-MO-A1*-VTX00029 | 1 | 454 | 0 | 0 | 0 | 0 | 0 | 3 | 0 | 0 | 0 | 0 |
|  |  | *Acaulospora-sp.*-VTX00231 | 1 | 0 | 0 | 0 | 0 | 0 | 0 | 0 | 0 | 0 | 0 | 0 |
|  |  | *Acaulospora-sp.*-VTX00328 | 1 | 0 | 0 | 0 | 0 | 0 | 0 | 0 | 0 | 0 | 0 | 0 |
|  |  | Unclassified *Acaulospora* | 8 | 206 | 0 | 0 | 0 | 0 | 0 | 8 | 0 | 0 | 0 | 0 |
| Diversisporaceae | *Diversispora* | *Diversispora-MO-GC1*-VTX00060 | 1 | 3 | 34 | 120 | 317 | 0 | 3 | 79 | 2 | 90 | 20 | 0 |
|  |  | *Diversispora-sp.*-VTX00058 | 1 | 0 | 0 | 0 | 0 | 0 | 0 | 0 | 0 | 0 | 0 | 0 |
|  |  | Unclassified *Diversispora* | 7 | 59 | 106 | 160 | 120 | 0 | 148 | 156 | 14 | 475 | 1 | 18 |
|  | Diversisporaceae *Glomus* | *Glomus-versiforme*-VTX00061 | 6 | 207 | 0 | 2 | 2 | 0 | 1 | 94 | 21 | 2 | 0 | 22 |
|  | Unclassified Diversisporaceae | Unclassified Diversisporaceae | 1 | 0 | 3 | 0 | 645 | 0 | 45 | 5 | 0 | 0 | 0 | 0 |
| Gigasporaceae | *Gigaspora* | *Gigaspora-decipiens*-VTX00039 | 1 | 35 | 0 | 0 | 0 | 0 | 40 | 11 | 0 | 0 | 0 | 0 |
|  |  | *Gigaspora-LER04*-VTX00318 | 1 | 0 | 0 | 0 | 0 | 0 | 0 | 0 | 0 | 0 | 0 | 0 |
|  | *Scutellospora* | *Scutellospora-Scut1*-VTX00049 | 1 | 0 | 0 | 0 | 0 | 0 | 0 | 16 | 0 | 0 | 0 | 0 |
|  |  | Unclassified *Scutellospora* | 2 | 0 | 5 | 0 | 0 | 0 | 0 | 2 | 0 | 0 | 0 | 0 |
|  | Unclassified Gigasporaceae | Unclassified Gigasporaceae | 2 | 1 | 0 | 0 | 0 | 0 | 10 | 1 | 0 | 0 | 0 | 0 |
| Glomeraceae | Glomeraceae *Glomus* | *Glomus-acnaGlo2*-VTX00155 | 2 | 25 | 451 | 1395 | 210 | 102 | 269 | 103 | 319 | 0 | 0 | 0 |
|  |  | *Glomus-caledonium*-VTX00065 | 4 | 550 | 3058 | 0 | 1 | 521 | 35 | 0 | 0 | 0 | 0 | 16 |
|  |  | *Glomus-clarum*-VTX00264 | 1 | 0 | 0 | 0 | 0 | 0 | 0 | 0 | 0 | 0 | 0 | 0 |
|  |  | *Glomus-GlAd2.2*-VTX00210 | 1 | 0 | 0 | 0 | 0 | 0 | 0 | 0 | 0 | 0 | 47 | 0 |
|  |  | *Glomus-GlAd3.3*-VTX00289 | 3 | 489 | 62 | 0 | 0 | 0 | 130 | 1053 | 1043 | 0 | 95 | 392 |
|  |  | *Glomus-Glo3b*-VTX00069 | 3 | 320 | 531 | 844 | 245 | 0 | 351 | 42 | 177 | 760 | 783 | 3983 |
|  |  | *Glomus-Glo7*-VTX00214 | 7 | 229 | 1254 | 1841 | 907 | 805 | 537 | 614 | 645 | 1643 | 42 | 158 |
|  |  | *Glomus-group-B-Glomus-acnaGlo7*-VTX00057 | 4 | 147 | 386 | 284 | 208 | 0 | 151 | 50 | 0 | 163 | 0 | 0 |
|  |  | *Glomus-group-B-Glomus-Douhan9*-VTX00056 | 2 | 141 | 575 | 0 | 0 | 0 | 0 | 0 | 22 | 51 | 352 | 78 |
|  |  | *Glomus-group-B-Glomus-GlBb1.2*-VTX00055 | 2 | 134 | 11 | 0 | 0 | 0 | 123 | 0 | 0 | 0 | 2480 | 155 |
|  |  | *Glomus-group-B-Glomus-Glo58*-VTX00225 | 3 | 1293 | 42 | 0 | 1 | 0 | 0 | 146 | 702 | 0 | 1307 | 15 |
|  |  | *Glomus-group-B-Glomus-Glo59*-VTX00276 | 2 | 2 | 0 | 0 | 0 | 0 | 0 | 444 | 0 | 0 | 771 | 82 |
|  |  | *Glomus-group-B-Glomus-Glo-G8*-VTX00340 | 2 | 1 | 23 | 5 | 30 | 0 | 4 | 1 | 2 | 2 | 0 | 0 |
|  |  | *Glomus-group-B-Glomus-lamellosu*-VTX00193 | 8 | 121 | 590 | 98 | 2927 | 502 | 541 | 133 | 322 | 53 | 248 | 298 |
|  |  | *Glomus-group-B-Glomus-ORVIN-GLO4*-VTX00278 | 3 | 236 | 3 | 110 | 0 | 0 | 0 | 516 | 969 | 0 | 0 | 0 |
|  |  | *Glomus-MO-G13*-VTX00115 | 1 | 7 | 65 | 167 | 0 | 0 | 0 | 46 | 74 | 139 | 0 | 30 |
|  |  | *Glomus-MO-G14*-VTX00083 | 3 | 0 | 0 | 0 | 0 | 0 | 0 | 0 | 0 | 0 | 434 | 0 |
|  |  | *Glomus-MO-G16*-VTX00072 | 3 | 0 | 31 | 0 | 0 | 0 | 0 | 2189 | 647 | 263 | 0 | 0 |
|  |  | *Glomus-MO-G17*-VTX00114 | 4 | 9 | 32 | 4 | 122 | 19 | 15 | 20 | 1 | 28 | 1 | 3 |
|  |  | *Glomus-MO-G18*-VTX00064 | 5 | 1528 | 2384 | 1764 | 1520 | 3353 | 3059 | 973 | 497 | 2318 | 476 | 233 |
|  |  | *Glomus-MO-G22*-VTX00125 | 2 | 173 | 133 | 0 | 0 | 0 | 118 | 96 | 14 | 40 | 19 | 88 |
|  |  | *Glomus-MO-G23*-VTX00222 | 1 | 93 | 78 | 1 | 185 | 410 | 205 | 35 | 0 | 54 | 108 | 93 |
|  |  | *Glomus-MO-G7*-VTX00199 | 2 | 0 | 0 | 0 | 0 | 0 | 0 | 0 | 0 | 0 | 0 | 0 |
|  |  | *Glomus-mosseae*-VTX00067 | 2 | 1 | 247 | 176 | 607 | 2 | 296 | 106 | 0 | 859 | 973 | 954 |
|  |  | *Glomus-ORVIN-GLO3B*-VTX00223 | 3 | 0 | 0 | 0 | 0 | 0 | 0 | 0 | 1145 | 0 | 81 | 0 |
|  |  | *Glomus-ORVIN-GLO3D*-VTX00310 | 3 | 281 | 0 | 0 | 0 | 0 | 0 | 0 | 3131 | 0 | 0 | 0 |
|  |  | *Glomus-ORVIN-GLO3E*-VTX00309 | 1 | 80 | 0 | 0 | 0 | 0 | 0 | 0 | 0 | 0 | 0 | 0 |
|  |  | *Glomus-perpusillum*-VTX00287 | 1 | 0 | 0 | 0 | 0 | 0 | 0 | 0 | 0 | 0 | 0 | 1 |
|  |  | *Glomus-sp.*-VTX00093 | 1 | 2 | 0 | 0 | 0 | 0 | 0 | 1 | 0 | 0 | 0 | 479 |
|  |  | *Glomus-sp.*-VTX00165 | 1 | 0 | 0 | 0 | 0 | 0 | 0 | 0 | 0 | 0 | 24 | 0 |
|  |  | *Glomus-sp.*-VTX00301 | 1 | 0 | 0 | 0 | 0 | 0 | 0 | 0 | 0 | 0 | 0 | 0 |
|  |  | *Glomus-sp.*-VTX00304 | 1 | 0 | 0 | 139 | 0 | 0 | 0 | 0 | 0 | 0 | 0 | 0 |
|  |  | *Glomus-sp.*-VTX00330 | 1 | 0 | 0 | 0 | 0 | 0 | 0 | 0 | 0 | 0 | 0 | 3 |
|  |  | *Glomus-viscosum*-VTX00063 | 6 | 110 | 429 | 360 | 25 | 864 | 423 | 0 | 124 | 1 | 0 | 4 |
|  |  | *Glomus-Wirsel-OTU12*-VTX00188 | 1 | 0 | 0 | 0 | 0 | 0 | 0 | 0 | 0 | 0 | 0 | 0 |
|  |  | *Glomus-Wirsel-OTU14*-VTX00137 | 1 | 274 | 253 | 229 | 270 | 0 | 385 | 238 | 50 | 154 | 14 | 12 |
|  |  | *Glomus-Wirsel-OTU16*-VTX00156 | 3 | 0 | 0 | 0 | 0 | 0 | 1 | 0 | 0 | 0 | 1 | 2 |
|  |  | *Glomus-Wirsel-OTU4*-VTX00086 | 1 | 0 | 0 | 10 | 0 | 0 | 0 | 0 | 0 | 0 | 0 | 0 |
|  |  | *Glomus-Wirsel-OTU6*-VTX00202 | 1 | 0 | 0 | 0 | 0 | 0 | 0 | 0 | 0 | 0 | 61 | 0 |
|  |  | *Glomus-Yamato08-A1*-VTX00100 | 2 | 0 | 0 | 0 | 0 | 0 | 0 | 0 | 0 | 0 | 10 | 3562 |
|  |  | *Glomus-Yamato09-C1*-VTX00174 | 1 | 0 | 0 | 0 | 0 | 0 | 0 | 274 | 0 | 0 | 14 | 0 |
|  |  | Unclassified Glomeraceae *Glomus* | 94 | 6802 | 4658 | 7818 | 7177 | 8984 | 8666 | 6718 | 4257 | 7861 | 5842 | 3448 |
| Paraglomeraceae | *Paraglomus* | *Paraglomus-brasilianum*-VTX00239 | 1 | 25 | 0 | 0 | 0 | 0 | 0 | 9 | 18 | 0 | 0 | 5 |
|  |  | *Paraglomus-Glom-1B.13*-VTX00308 | 2 | 0 | 0 | 0 | 0 | 0 | 0 | 0 | 0 | 0 | 0 | 0 |
|  |  | *Paraglomus-Para1-OTU2*-VTX00337 | 2 | 1476 | 73 | 0 | 0 | 0 | 6 | 1133 | 1360 | 498 | 968 | 1327 |
|  |  | *Paraglomus-Para1*-VTX00336 | 1 | 0 | 0 | 0 | 0 | 0 | 0 | 0 | 0 | 0 | 0 | 0 |
|  |  | Unclassified *Paraglomus* | 2 | 4 | 0 | 0 | 43 | 0 | 0 | 155 | 0 | 108 | 390 | 87 |
| Unclassified Glomeromycetes | Unclassified Glomeromycetes | Unclassified Glomeromycetes | 19 | 8 | 45 | 35 | 0 | 0 | 0 | 7 | 2 | 0 | 0 | 12 |
|  |  | Total | 268 | 15562 | 15562 | 15562 | 15562 | 15562 | 15562 | 15562 | 15562 | 15562 | 15562 | 15562 |
| Family | Genus | Species |  | S1-Bn1 | S1-Aa2 | S1-Ra2 | S1-Bn2 | S1-Aa3 | S1-Ra3 | S1-Bn3 | S1-Aa4 | S1-Ra4 | S1-Bn4 | S2-Aa1 |
| Archaeosporaceae | *Archaeospora* | *Archaeospora-Aca*-VTX00338 |  | 0 | 0 | 0 | 0 | 1 | 0 | 7 | 2 | 0 | 0 | 0 |
|  |  | *Archaeospora-Other1*-VTX00005 |  | 0 | 0 | 0 | 0 | 0 | 1 | 1 | 2 | 0 | 0 | 0 |
|  |  | *Archaeospora-sp.*-VTX00009 |  | 0 | 0 | 0 | 0 | 1 | 2 | 2 | 3 | 0 | 0 | 0 |
|  |  | *Archaeospora-trappei*-VTX00245 |  | 0 | 0 | 0 | 0 | 0 | 0 | 0 | 1 | 0 | 0 | 0 |
|  |  | Unclassified *Archaeospora* |  | 0 | 0 | 0 | 0 | 0 | 52 | 4 | 24 | 0 | 0 | 0 |
|  | norank | *unknown-Wirsel-OUT21*-VTX00004 |  | 0 | 0 | 0 | 0 | 0 | 0 | 6 | 0 | 0 | 0 | 0 |
| Acaulosporaceae | *Acaulospora* | *Acaulospora-Acau11*-VTX00031 |  | 0 | 0 | 0 | 0 | 0 | 0 | 0 | 71 | 0 | 0 | 0 |
|  |  | *Acaulospora-Acau12*-VTX00045 |  | 0 | 0 | 0 | 0 | 0 | 0 | 0 | 2247 | 28 | 17 | 0 |
|  |  | *Acaulospora-Acau8*-VTX00027 |  | 0 | 0 | 0 | 0 | 0 | 0 | 7 | 0 | 0 | 5 | 0 |
|  |  | *Acaulospora-MO-A1*-VTX00029 |  | 0 | 140 | 0 | 0 | 27 | 0 | 0 | 35 | 0 | 0 | 0 |
|  |  | *Acaulospora-sp.*-VTX00231 |  | 0 | 0 | 0 | 0 | 0 | 0 | 0 | 56 | 0 | 0 | 0 |
|  |  | *Acaulospora-sp.*-VTX00328 |  | 0 | 0 | 0 | 0 | 0 | 0 | 0 | 35 | 0 | 0 | 0 |
|  |  | Unclassified *Acaulospora* |  | 0 | 0 | 84 | 0 | 8 | 0 | 164 | 1397 | 2284 | 72 | 0 |
| Diversisporaceae | *Diversispora* | *Diversispora-MO-GC1*-VTX00060 |  | 0 | 243 | 136 | 117 | 15 | 0 | 11 | 3 | 326 | 2 | 2 |
|  |  | *Diversispora-sp.*-VTX00058 |  | 0 | 0 | 0 | 0 | 0 | 0 | 0 | 0 | 0 | 0 | 0 |
|  |  | Unclassified *Diversispora* |  | 5 | 548 | 145 | 795 | 111 | 30 | 903 | 544 | 2127 | 213 | 12 |
|  | Diversisporaceae *Glomus* | *Glomus-versiforme*-VTX00061 |  | 21 | 47 | 2 | 16 | 115 | 5 | 1 | 0 | 96 | 0 | 25 |
|  | Unclassified Diversisporaceae | Unclassified Diversisporaceae |  | 3 | 124 | 21 | 48 | 28 | 0 | 0 | 0 | 146 | 0 | 0 |
| Gigasporaceae | *Gigaspora* | *Gigaspora-decipiens*-VTX00039 |  | 0 | 0 | 0 | 0 | 0 | 0 | 309 | 0 | 0 | 0 | 0 |
|  |  | *Gigaspora-LER04*-VTX00318 |  | 0 | 0 | 0 | 0 | 0 | 0 | 0 | 208 | 0 | 0 | 0 |
|  | *Scutellospora* | *Scutellospora-Scut1*-VTX00049 |  | 0 | 0 | 0 | 0 | 0 | 0 | 0 | 18 | 0 | 0 | 0 |
|  |  | Unclassified *Scutellospora* |  | 0 | 0 | 0 | 0 | 75 | 0 | 9 | 0 | 0 | 0 | 0 |
|  | Unclassified Gigasporaceae | Unclassified Gigasporaceae |  | 0 | 0 | 0 | 0 | 0 | 0 | 112 | 275 | 0 | 0 | 0 |
| Glomeraceae | Glomeraceae *Glomus* | *Glomus-acnaGlo2*-VTX00155 |  | 0 | 0 | 0 | 0 | 0 | 0 | 35 | 1 | 3 | 0 | 35 |
|  |  | *Glomus-caledonium*-VTX00065 |  | 894 | 0 | 2316 | 4 | 0 | 0 | 2373 | 0 | 0 | 9501 | 0 |
|  |  | *Glomus-clarum*-VTX00264 |  | 0 | 0 | 0 | 0 | 0 | 169 | 0 | 0 | 0 | 0 | 0 |
|  |  | *Glomus-GlAd2.2*-VTX00210 |  | 0 | 0 | 0 | 0 | 0 | 0 | 0 | 0 | 0 | 0 | 0 |
|  |  | *Glomus-GlAd3.3*-VTX00289 |  | 0 | 0 | 0 | 0 | 326 | 284 | 1184 | 778 | 0 | 0 | 0 |
|  |  | *Glomus-Glo3b*-VTX00069 |  | 10 | 48 | 10 | 61 | 313 | 154 | 65 | 87 | 271 | 0 | 0 |
|  |  | *Glomus-Glo7*-VTX00214 |  | 59 | 167 | 49 | 161 | 453 | 61 | 105 | 17 | 48 | 0 | 133 |
|  |  | *Glomus-group-B-Glomus-acnaGlo7*-VTX00057 |  | 0 | 40 | 38 | 60 | 16 | 3 | 99 | 0 | 8 | 0 | 0 |
|  |  | *Glomus-group-B-Glomus-Douhan9*-VTX00056 |  | 49 | 1374 | 1846 | 2108 | 178 | 0 | 433 | 58 | 102 | 0 | 0 |
|  |  | *Glomus-group-B-Glomus-GlBb1.2*-VTX00055 |  | 7109 | 3876 | 199 | 203 | 631 | 0 | 126 | 0 | 324 | 0 | 498 |
|  |  | *Glomus-group-B-Glomus-Glo58*-VTX00225 |  | 0 | 0 | 0 | 0 | 1 | 0 | 351 | 1918 | 0 | 0 | 0 |
|  |  | *Glomus-group-B-Glomus-Glo59*-VTX00276 |  | 14 | 3009 | 5289 | 0 | 342 | 0 | 1 | 0 | 1856 | 2 | 180 |
|  |  | *Glomus-group-B-Glomus-Glo-G8*-VTX00340 |  | 0 | 0 | 0 | 0 | 0 | 14 | 0 | 0 | 0 | 0 | 0 |
|  |  | *Glomus-group-B-Glomus-lamellosu*-VTX00193 |  | 19 | 752 | 569 | 305 | 1153 | 483 | 230 | 565 | 426 | 39 | 2165 |
|  |  | *Glomus-group-B-Glomus-ORVIN-GLO4*-VTX00278 |  | 0 | 0 | 0 | 0 | 0 | 0 | 1 | 0 | 0 | 0 | 0 |
|  |  | *Glomus-MO-G13*-VTX00115 |  | 7 | 0 | 136 | 58 | 0 | 14 | 0 | 31 | 183 | 10 | 0 |
|  |  | *Glomus-MO-G14*-VTX00083 |  | 7 | 0 | 0 | 0 | 0 | 0 | 0 | 0 | 0 | 0 | 0 |
|  |  | *Glomus-MO-G16*-VTX00072 |  | 0 | 0 | 0 | 0 | 0 | 0 | 0 | 0 | 0 | 0 | 0 |
|  |  | *Glomus-MO-G17*-VTX00114 |  | 1 | 313 | 38 | 68 | 94 | 0 | 1 | 3 | 14 | 3 | 674 |
|  |  | *Glomus-MO-G18*-VTX00064 |  | 333 | 727 | 110 | 553 | 1867 | 608 | 447 | 196 | 311 | 232 | 49 |
|  |  | *Glomus-MO-G22*-VTX00125 |  | 17 | 0 | 128 | 436 | 406 | 30 | 67 | 0 | 465 | 399 | 23 |
|  |  | *Glomus-MO-G23*-VTX00222 |  | 125 | 0 | 271 | 592 | 841 | 34 | 49 | 291 | 1706 | 2472 | 12 |
|  |  | *Glomus-MO-G7*-VTX00199 |  | 0 | 0 | 0 | 0 | 0 | 0 | 122 | 175 | 40 | 0 | 0 |
|  |  | *Glomus-mosseae*-VTX00067 |  | 5943 | 1 | 34 | 3987 | 559 | 0 | 4458 | 0 | 0 | 67 | 6025 |
|  |  | *Glomus-ORVIN-GLO3B*-VTX00223 |  | 0 | 0 | 0 | 0 | 0 | 0 | 0 | 2076 | 0 | 0 | 0 |
|  |  | *Glomus-ORVIN-GLO3D*-VTX00310 |  | 0 | 0 | 0 | 0 | 0 | 0 | 0 | 0 | 0 | 0 | 0 |
|  |  | *Glomus-ORVIN-GLO3E*-VTX00309 |  | 0 | 0 | 0 | 0 | 0 | 339 | 0 | 0 | 0 | 0 | 14 |
|  |  | *Glomus-perpusillum*-VTX00287 |  | 0 | 0 | 0 | 0 | 150 | 0 | 69 | 0 | 0 | 0 | 62 |
|  |  | *Glomus-sp.*-VTX00093 |  | 0 | 0 | 0 | 0 | 0 | 1063 | 0 | 15 | 0 | 0 | 0 |
|  |  | *Glomus-sp.*-VTX00165 |  | 29 | 299 | 0 | 21 | 250 | 0 | 0 | 0 | 0 | 11 | 260 |
|  |  | *Glomus-sp.*-VTX00301 |  | 0 | 34 | 0 | 0 | 0 | 0 | 0 | 0 | 10 | 0 | 25 |
|  |  | *Glomus-sp.*-VTX00304 |  | 0 | 94 | 0 | 0 | 0 | 0 | 0 | 0 | 365 | 0 | 84 |
|  |  | *Glomus-sp.*-VTX00330 |  | 0 | 0 | 0 | 0 | 0 | 0 | 4 | 0 | 0 | 0 | 0 |
|  |  | *Glomus-viscosum*-VTX00063 |  | 18 | 0 | 29 | 13 | 26 | 0 | 63 | 0 | 0 | 0 | 209 |
|  |  | *Glomus-Wirsel-OTU12*-VTX00188 |  | 0 | 0 | 0 | 0 | 25 | 0 | 0 | 0 | 0 | 0 | 70 |
|  |  | *Glomus-Wirsel-OTU14*-VTX00137 |  | 8 | 0 | 12 | 21 | 0 | 0 | 0 | 24 | 30 | 0 | 0 |
|  |  | *Glomus-Wirsel-OTU16*-VTX00156 |  | 54 | 1 | 12 | 0 | 55 | 0 | 0 | 0 | 0 | 45 | 620 |
|  |  | *Glomus-Wirsel-OTU4*-VTX00086 |  | 0 | 0 | 4 | 0 | 0 | 0 | 0 | 0 | 0 | 0 | 2 |
|  |  | *Glomus-Wirsel-OTU6*-VTX00202 |  | 0 | 0 | 0 | 0 | 0 | 0 | 0 | 0 | 0 | 0 | 0 |
|  |  | *Glomus-Yamato08-A1*-VTX00100 |  | 0 | 101 | 0 | 0 | 50 | 155 | 0 | 0 | 0 | 0 | 10 |
|  |  | *Glomus-Yamato09-C1*-VTX00174 |  | 0 | 74 | 0 | 0 | 0 | 7 | 0 | 9 | 36 | 0 | 0 |
|  |  | Unclassified Glomeraceae *Glomus* |  | 828 | 3350 | 4070 | 5914 | 7359 | 5864 | 3182 | 3923 | 4077 | 2472 | 4372 |
| Paraglomeraceae | *Paraglomus* | *Paraglomus-brasilianum*-VTX00239 |  | 0 | 0 | 0 | 0 | 2 | 458 | 38 | 17 | 0 | 0 | 0 |
|  |  | *Paraglomus-Glom-1B.13*-VTX00308 |  | 0 | 0 | 0 | 2 | 0 | 0 | 2 | 0 | 0 | 0 | 0 |
|  |  | *Paraglomus-Para1-OTU2*-VTX00337 |  | 7 | 0 | 7 | 12 | 67 | 5232 | 491 | 442 | 22 | 0 | 1 |
|  |  | *Paraglomus-Para1*-VTX00336 |  | 0 | 0 | 1 | 6 | 0 | 0 | 0 | 0 | 0 | 0 | 0 |
|  |  | Unclassified *Paraglomus* |  | 2 | 11 | 6 | 0 | 2 | 456 | 0 | 8 | 242 | 0 | 0 |
| Unclassified Glomeromycetes | Unclassified Glomeromycetes | Unclassified Glomeromycetes |  | 0 | 189 | 0 | 1 | 15 | 44 | 30 | 7 | 16 | 0 | 0 |
|  |  | Total |  | 15562 | 15562 | 15562 | 15562 | 15562 | 15562 | 15562 | 15562 | 15562 | 15562 | 15562 |
| Family | Genus | Species |  | S2-Rn1 | S2-Bl1 | S2-Aa2 | S2-Rn2 | S2-Bl2 | S2-Aa3 | S2-Rn3 | S2-Bl3 | S2-Aa4 | S2-Rn4 | S2-Bl4 |
| Archaeosporaceae | *Archaeospora* | *Archaeospora-Aca*-VTX00338 |  | 0 | 0 | 0 | 0 | 0 | 0 | 0 | 0 | 0 | 0 | 0 |
|  |  | *Archaeospora-Other1*-VTX00005 |  | 0 | 0 | 0 | 0 | 0 | 0 | 0 | 0 | 0 | 0 | 0 |
|  |  | *Archaeospora-sp.*-VTX00009 |  | 0 | 0 | 0 | 0 | 0 | 0 | 0 | 0 | 0 | 0 | 0 |
|  |  | *Archaeospora-trappei*-VTX00245 |  | 0 | 0 | 0 | 0 | 0 | 0 | 0 | 0 | 0 | 0 | 0 |
|  |  | Unclassified *Archaeospora* |  | 0 | 0 | 0 | 0 | 0 | 0 | 0 | 0 | 0 | 0 | 0 |
|  | norank | *unknown-Wirsel-OUT21*-VTX00004 |  | 0 | 0 | 0 | 0 | 0 | 0 | 0 | 0 | 0 | 0 | 0 |
| Acaulosporaceae | *Acaulospora* | *Acaulospora-Acau11*-VTX00031 |  | 0 | 0 | 0 | 0 | 0 | 0 | 0 | 0 | 0 | 0 | 0 |
|  |  | *Acaulospora-Acau12*-VTX00045 |  | 0 | 0 | 0 | 0 | 0 | 24 | 0 | 0 | 0 | 0 | 0 |
|  |  | *Acaulospora-Acau8*-VTX00027 |  | 0 | 68 | 0 | 0 | 0 | 0 | 0 | 0 | 0 | 0 | 0 |
|  |  | *Acaulospora-MO-A1*-VTX00029 |  | 0 | 8 | 0 | 0 | 0 | 0 | 0 | 0 | 0 | 0 | 0 |
|  |  | *Acaulospora-sp.*-VTX00231 |  | 0 | 0 | 0 | 0 | 0 | 0 | 0 | 0 | 0 | 0 | 0 |
|  |  | *Acaulospora-sp.*-VTX00328 |  | 0 | 0 | 0 | 0 | 0 | 0 | 0 | 0 | 0 | 0 | 0 |
|  |  | Unclassified *Acaulospora* |  | 45 | 0 | 0 | 0 | 0 | 2 | 0 | 0 | 0 | 0 | 0 |
| Diversisporaceae | *Diversispora* | *Diversispora-MO-GC1*-VTX00060 |  | 33 | 4 | 0 | 0 | 0 | 0 | 0 | 15 | 0 | 971 | 0 |
|  |  | *Diversispora-sp.*-VTX00058 |  | 11 | 0 | 158 | 0 | 0 | 0 | 0 | 0 | 0 | 0 | 0 |
|  |  | Unclassified *Diversispora* |  | 152 | 93 | 44 | 0 | 0 | 0 | 0 | 73 | 1005 | 5 | 0 |
|  | Diversisporaceae *Glomus* | *Glomus-versiforme*-VTX00061 |  | 325 | 37 | 1 | 0 | 0 | 0 | 0 | 165 | 7903 | 6315 | 3873 |
|  | Unclassified Diversisporaceae | Unclassified Diversisporaceae |  | 3 | 1 | 0 | 0 | 0 | 0 | 0 | 0 | 2 | 144 | 0 |
| Gigasporaceae | *Gigaspora* | *Gigaspora-decipiens*-VTX00039 |  | 0 | 0 | 0 | 0 | 0 | 0 | 0 | 0 | 0 | 0 | 0 |
|  |  | *Gigaspora-LER04*-VTX00318 |  | 0 | 0 | 0 | 0 | 0 | 0 | 0 | 0 | 0 | 0 | 0 |
|  | *Scutellospora* | *Scutellospora-Scut1*-VTX00049 |  | 0 | 0 | 0 | 0 | 0 | 0 | 0 | 0 | 0 | 0 | 0 |
|  |  | Unclassified *Scutellospora* |  | 0 | 0 | 0 | 0 | 0 | 0 | 0 | 0 | 0 | 0 | 0 |
|  | Unclassified Gigasporaceae | Unclassified Gigasporaceae |  | 0 | 0 | 0 | 0 | 0 | 0 | 0 | 0 | 0 | 0 | 0 |
| Glomeraceae | Glomeraceae *Glomus* | *Glomus-acnaGlo2*-VTX00155 |  | 0 | 132 | 0 | 0 | 0 | 0 | 0 | 0 | 0 | 0 | 0 |
|  |  | *Glomus-caledonium*-VTX00065 |  | 0 | 0 | 1 | 0 | 0 | 0 | 0 | 0 | 0 | 0 | 0 |
|  |  | *Glomus-clarum*-VTX00264 |  | 0 | 0 | 0 | 0 | 0 | 0 | 0 | 0 | 0 | 0 | 0 |
|  |  | *Glomus-GlAd2.2*-VTX00210 |  | 0 | 0 | 0 | 0 | 0 | 0 | 0 | 0 | 0 | 0 | 0 |
|  |  | *Glomus-GlAd3.3*-VTX00289 |  | 0 | 0 | 0 | 0 | 0 | 0 | 0 | 0 | 0 | 0 | 0 |
|  |  | *Glomus-Glo3b*-VTX00069 |  | 0 | 52 | 0 | 0 | 0 | 0 | 0 | 0 | 0 | 0 | 0 |
|  |  | *Glomus-Glo7*-VTX00214 |  | 140 | 1025 | 270 | 53 | 1272 | 0 | 0 | 94 | 0 | 0 | 0 |
|  |  | *Glomus-group-B-Glomus-acnaGlo7*-VTX00057 |  | 0 | 0 | 0 | 0 | 0 | 0 | 0 | 187 | 0 | 0 | 0 |
|  |  | *Glomus-group-B-Glomus-Douhan9*-VTX00056 |  | 0 | 0 | 56 | 0 | 0 | 0 | 0 | 4745 | 0 | 0 | 0 |
|  |  | *Glomus-group-B-Glomus-GlBb1.2*-VTX00055 |  | 2534 | 1864 | 414 | 1333 | 0 | 12 | 0 | 59 | 0 | 0 | 5559 |
|  |  | *Glomus-group-B-Glomus-Glo58*-VTX00225 |  | 0 | 0 | 0 | 0 | 0 | 0 | 0 | 0 | 0 | 0 | 0 |
|  |  | *Glomus-group-B-Glomus-Glo59*-VTX00276 |  | 3777 | 104 | 98 | 1493 | 0 | 0 | 0 | 162 | 0 | 0 | 0 |
|  |  | *Glomus-group-B-Glomus-Glo-G8*-VTX00340 |  | 0 | 0 | 0 | 0 | 0 | 0 | 0 | 0 | 0 | 0 | 0 |
|  |  | *Glomus-group-B-Glomus-lamellosu*-VTX00193 |  | 0 | 305 | 464 | 1043 | 0 | 161 | 0 | 1086 | 6644 | 0 | 0 |
|  |  | *Glomus-group-B-Glomus-ORVIN-GLO4*-VTX00278 |  | 0 | 0 | 1 | 0 | 0 | 0 | 0 | 0 | 0 | 0 | 0 |
|  |  | *Glomus-MO-G13*-VTX00115 |  | 0 | 0 | 0 | 0 | 0 | 0 | 0 | 0 | 0 | 0 | 0 |
|  |  | *Glomus-MO-G14*-VTX00083 |  | 0 | 0 | 0 | 0 | 0 | 0 | 0 | 0 | 0 | 0 | 0 |
|  |  | *Glomus-MO-G16*-VTX00072 |  | 0 | 1 | 0 | 0 | 0 | 0 | 0 | 0 | 0 | 0 | 0 |
|  |  | *Glomus-MO-G17*-VTX00114 |  | 1479 | 418 | 549 | 373 | 0 | 170 | 344 | 197 | 0 | 1933 | 0 |
|  |  | *Glomus-MO-G18*-VTX00064 |  | 72 | 699 | 0 | 0 | 0 | 0 | 0 | 4284 | 0 | 0 | 5587 |
|  |  | *Glomus-MO-G22*-VTX00125 |  | 1 | 59 | 0 | 0 | 0 | 0 | 0 | 0 | 0 | 0 | 0 |
|  |  | *Glomus-MO-G23*-VTX00222 |  | 0 | 226 | 0 | 0 | 0 | 16 | 0 | 0 | 0 | 0 | 0 |
|  |  | *Glomus-MO-G7*-VTX00199 |  | 0 | 0 | 0 | 0 | 0 | 0 | 0 | 0 | 0 | 0 | 0 |
|  |  | *Glomus-mosseae*-VTX00067 |  | 344 | 1534 | 137 | 4784 | 0 | 13291 | 14570 | 0 | 0 | 0 | 0 |
|  |  | *Glomus-ORVIN-GLO3B*-VTX00223 |  | 0 | 0 | 0 | 0 | 0 | 0 | 0 | 0 | 0 | 0 | 0 |
|  |  | *Glomus-ORVIN-GLO3D*-VTX00310 |  | 0 | 0 | 0 | 0 | 0 | 0 | 0 | 0 | 0 | 0 | 0 |
|  |  | *Glomus-ORVIN-GLO3E*-VTX00309 |  | 0 | 0 | 1725 | 0 | 0 | 0 | 0 | 0 | 0 | 0 | 0 |
|  |  | *Glomus-perpusillum*-VTX00287 |  | 0 | 187 | 75 | 0 | 3734 | 0 | 0 | 517 | 0 | 0 | 0 |
|  |  | *Glomus-sp.*-VTX00093 |  | 0 | 0 | 0 | 0 | 0 | 0 | 0 | 0 | 0 | 0 | 0 |
|  |  | *Glomus-sp.*-VTX00165 |  | 188 | 339 | 0 | 0 | 0 | 528 | 0 | 0 | 0 | 0 | 0 |
|  |  | *Glomus-sp.*-VTX00301 |  | 0 | 10 | 520 | 0 | 0 | 0 | 0 | 0 | 0 | 0 | 0 |
|  |  | *Glomus-sp.*-VTX00304 |  | 516 | 62 | 2853 | 0 | 0 | 0 | 0 | 0 | 0 | 0 | 0 |
|  |  | *Glomus-sp.*-VTX00330 |  | 0 | 0 | 0 | 0 | 0 | 0 | 0 | 0 | 0 | 0 | 0 |
|  |  | *Glomus-viscosum*-VTX00063 |  | 59 | 190 | 0 | 0 | 0 | 0 | 0 | 0 | 0 | 0 | 0 |
|  |  | *Glomus-Wirsel-OTU12*-VTX00188 |  | 73 | 55 | 0 | 0 | 0 | 0 | 0 | 0 | 0 | 0 | 0 |
|  |  | *Glomus-Wirsel-OTU14*-VTX00137 |  | 1 | 0 | 72 | 0 | 0 | 0 | 0 | 0 | 0 | 0 | 0 |
|  |  | *Glomus-Wirsel-OTU16*-VTX00156 |  | 650 | 699 | 394 | 1422 | 0 | 223 | 0 | 67 | 0 | 2117 | 0 |
|  |  | *Glomus-Wirsel-OTU4*-VTX00086 |  | 11 | 10 | 0 | 0 | 0 | 0 | 0 | 0 | 0 | 0 | 0 |
|  |  | *Glomus-Wirsel-OTU6*-VTX00202 |  | 0 | 0 | 0 | 0 | 0 | 0 | 0 | 0 | 0 | 0 | 0 |
|  |  | *Glomus-Yamato08-A1*-VTX00100 |  | 56 | 0 | 2453 | 0 | 0 | 0 | 0 | 177 | 0 | 0 | 0 |
|  |  | *Glomus-Yamato09-C1*-VTX00174 |  | 80 | 0 | 0 | 0 | 0 | 0 | 0 | 64 | 0 | 0 | 0 |
|  |  | Unclassified Glomeraceae *Glomus* |  | 5004 | 7378 | 4643 | 5058 | 6928 | 1130 | 629 | 3525 | 8 | 4077 | 543 |
| Paraglomeraceae | *Paraglomus* | *Paraglomus-brasilianum*-VTX00239 |  | 0 | 0 | 3 | 0 | 3557 | 0 | 0 | 0 | 0 | 0 | 0 |
|  |  | *Paraglomus-Glom-1B.13*-VTX00308 |  | 0 | 0 | 0 | 0 | 0 | 0 | 0 | 0 | 0 | 0 | 0 |
|  |  | *Paraglomus-Para1-OTU2*-VTX00337 |  | 0 | 1 | 459 | 0 | 0 | 5 | 0 | 133 | 0 | 0 | 0 |
|  |  | *Paraglomus-Para1*-VTX00336 |  | 3 | 0 | 0 | 0 | 0 | 0 | 0 | 0 | 0 | 0 | 0 |
|  |  | Unclassified *Paraglomus* |  | 0 | 1 | 155 | 0 | 0 | 0 | 0 | 12 | 0 | 0 | 0 |
| Unclassified Glomeromycetes | Unclassified Glomeromycetes | Unclassified Glomeromycetes |  | 5 | 0 | 17 | 3 | 71 | 0 | 19 | 0 | 0 | 0 | 0 |
|  |  | Total |  | 15562 | 15562 | 15562 | 15562 | 15562 | 15562 | 15562 | 15562 | 15562 | 15562 | 15562 |
