# Supplementary material for: Arbuscular mycorrhizal fungi community analysis revealed the significant impact of arsenic in antimony- and arsenic-contaminated soil in three Guizhou regions
Source: Front Microbiol. 2023 May 18;14:1189400. doi: 10.3389/fmicb.2023.1189400 (PMC10232906; doi:10.3389/fmicb.2023.1189400)
Supplement: Supplementary file 17 [file Table_5.docx]

**Supplementary Table** **5.** Permutational multivariate analysis of variance (PERMANOVA, plant and sampling sites) and analysis of similarities (ANOSIM, pairwise comparison of sampling sites) showing the Bray–Curtis distance-based dissimilarity of arbuscular mycorrhizal fungal communities among the three sampling sites.

| Group | SS | MS | F model | R^2^ | *p* value |
| --- | --- | --- | --- | --- | --- |
| Plant | 1.867 | 0.467 | 1.362 | 0.163 | 0.028 |
| Sampling sites | 2.163 | 1.082 | 3.492 | 0.189 | 0.001 |
| S0 vs S1 | 1.001 | 1.001 | 3.583 | 0.159 | 0.001 |
| S0 vs S2 | 1.394 | 1.394 | 4.654 | 0.197 | 0.001 |
| S1 vs S2 | 0.879 | 0.879 | 2.549 | 0.104 | 0.001 |

Note: SS: Sum of squares; MS: Mean square.
