# Supplementary material for: Arbuscular mycorrhizal fungi community analysis revealed the significant impact of arsenic in antimony- and arsenic-contaminated soil in three Guizhou regions
Source: Front Microbiol. 2023 May 18;14:1189400. doi: 10.3389/fmicb.2023.1189400 (PMC10232906; doi:10.3389/fmicb.2023.1189400)
Supplement: Supplementary file 18 [file Table_6.docx]

**Supplementary Table 6.** Soil properties before and after multivariate collinear screening (variance inflation factor, VIF > 10).

| Before multivariate collinear screening | | | | | | | | | | | |
| --- | --- | --- | --- | --- | --- | --- | --- | --- | --- | --- | --- |
| Index | AN | AP | AK | TOC | pH | EC | TCa | TSb | DTPA-Sb | TAs | DTPA-As |
| VIF | 7.607 | 9.108 | 3.311 | 5.340 | 3.432 | 10.977 | 8.756 | 4.065 | 6.372 | 19.983 | 3.081 |
| After multivariate collinear screening | | | | | | | | | | | |
| Index | AN | AK | TOC | pH | TCa | TSb | DTPA-Sb | TAs | DTPA-As |  |  |
| VIF | 5.931 | 3.139 | 4.706 | 3.106 | 6.186 | 3.133 | 5.185 | 6.227 | 2.366 |  |  |

Note: AN: available nitrogen; AP: available phosphorus; AK: available potassium; TOC: total organic carbon; TSb: total antimony; DTPA-Sb: diethylenetriamine pentaacetic acid -extractable antimony; TAs: total arsenic; DTPA-As: diethylenetriamine pentaacetic acid -extractable antimony; EC: electrical conductivity; TCa: total calcium.
