# Supplementary material for: Arbuscular mycorrhizal fungi community analysis revealed the significant impact of arsenic in antimony- and arsenic-contaminated soil in three Guizhou regions
Source: Front Microbiol. 2023 May 18;14:1189400. doi: 10.3389/fmicb.2023.1189400 (PMC10232906; doi:10.3389/fmicb.2023.1189400)
Supplement: Supplementary file 19 [file Table_7.docx]

**Supplementary Table 7.** Correlation coefficients, R^2^, and significance values for soil properties with canonical correspondence analysis (CCA) axes from the sampling sites (Fig. 3).

|  | CCA1 | CCA2 | R^2^ | *p* value |
| --- | --- | --- | --- | --- |
| AN | –0.9175 | 0.3978 | 0.4854 | **0.001** |
| AK | –0.4628 | 0.8864 | 0.4115 | **0.001** |
| TOC | –0.9766 | 0.2150 | 0.3700 | **0.001** |
| pH | 0.6623 | 0.7492 | 0.7831 | **0.001** |
| TCa | 1.0000 | –0.0093 | 0.4043 | **0.001** |
| TSb | 0.9783 | –0.2070 | 0.3231 | **0.007** |
| DTPA-Sb | 0.9974 | 0.0727 | 0.2347 | **0.030** |
| TAs | 0.8167 | –0.5771 | 0.6695 | **0.001** |
| DTPA-As | 0.8520 | 0.5235 | 0.0613 | 0.362 |

AN: available nitrogen; AK: available potassium; TOC: total organic carbon; TCa: total calcium; TSb: total antimony; DTPA-Sb: diethylenetriamine pentaacetic acid -extractable antimony; TAs: total arsenic; DTPA-As: diethylenetriamine pentaacetic acid -extractable antimony.
