# Supplementary material for: Arbuscular mycorrhizal fungi community analysis revealed the significant impact of arsenic in antimony- and arsenic-contaminated soil in three Guizhou regions
Source: Front Microbiol. 2023 May 18;14:1189400. doi: 10.3389/fmicb.2023.1189400 (PMC10232906; doi:10.3389/fmicb.2023.1189400)
Supplement: Supplementary file 20 [file Table_8.docx]

**Supplementary Table 8.** Pearson correlations between soil properties and arbuscular mycorrhizal fungi (AMF) colonization rate and spore density using canonical correspondence analysis.

| Index | Group | | AN | AP | AK | TOC | pH | EC | TCa | TSb | DTPA-Sb | TAs | DTPA-As |
| --- | --- | --- | --- | --- | --- | --- | --- | --- | --- | --- | --- | --- | --- |
| AMF colonization rate | All | | −0.060  0.741 | **0.356**  **0.042^*^** | −0.081  0.655 | −0.054  0.765 | −0.091 0.613 | 0.244 0.171 | 0.038 0.833 | 0.296 0.094 | 0.203 0.257 | 0.246 0.168 | 0.003 0.985 |
|  | Aa | | 0.301  0.369 | 0.305  0.361 | 0.209  0.537 | 0.453  0.162 | −0.351 0.291 | 0.370 0.263 | −0.241 0.474 | 0.423 0.195 | 0.147 0.667 | 0.088 0.798 | −0.145 0.671 |
|  | Ra | | 0.418  0.200 | 0.193  0.570 | 0.199  0.557 | 0.309  0.355 | −0.217 0.521 | −0.042 0.902 | −0.070 0.837 | 0.512 0.108 | 0.022 0.949 | 0.054 0.875 | −0.392 0.234 |
| Spore density | | **-** | **0.675**  **<0.001^***^** | −0.333  0.058 | **0.608**  **<0.001^***^** | **0.473**  **0.005^**^** | 0.222 0.214 | **−0.459 0.007^**^** | 0.012 0.948 | 0.032 0.859 | 0.108 0.550 | −0.267 0.134 | 0.258 0.147 |
| Sobs index on the OTU level | | **-** | **0.415**  **0.016^*^** | **−0.620**  **<0.001^***^** | 0.236  0.186 | 0.290  0.101 | −0.037 0.837 | **−0.643**  **<0.001^***^** | −0.283 0.110 | **−0.431 0.012^*^** | **−0.385 0.027^*^** | **−0.562 0.001^**^** | −0.135 0.453 |
| Shannon index on the OTU level | | **-** | 0.316  0.073 | **−0.659**  **<0.001^***^** | 0.180  0.316 | 0.271  0.127 | −0.177 0.326 | **−0.600**  **<0.001^***^** | **−0.409 0.018^*^** | **−0.514 0.002^**^** | **−0.488 0.004^**^** | **−0.583**  **<0.001^***^** | −0.145 0.420 |
| Shannoneven index on the OTU level | | **-** | 0.172  0.340 | **−0.545**  **0.001^**^** | 0.058  0.747 | 0.185  0.302 | −0.255 0.152 | **−0.415 0.016^*^** | **−0.414 0.017^*^** | **−0.421 0.015^*^** | **−0.481**  **0.005^**^** | **−0.461 0.007^**^** | 0.149 0.408 |

Note: The data for each cell are shown as Pearson's R (upper data) and p-value (lower data). Significance levels: *****, *p* < 0.05; ******, *p* < 0.01; *******, *p* < 0.001. All: Coefficient of correlation between AMF colonization rate and soil indexes of all samples; Aa: Coefficient of correlation between AMF colonization rate and soil indexes of *Artemisia argyi*. Ra: Coefficient of correlation between AMF colonization rate and soil indexes of *Rumex acetosa*. OTU: operational taxonomic unit.
