# Supplementary material for: Arbuscular mycorrhizal fungi community analysis revealed the significant impact of arsenic in antimony- and arsenic-contaminated soil in three Guizhou regions
Source: Front Microbiol. 2023 May 18;14:1189400. doi: 10.3389/fmicb.2023.1189400 (PMC10232906; doi:10.3389/fmicb.2023.1189400)
Supplement: Supplementary file 21 [file Table_9.docx]

**Supplementary Table 9** Pearson correlations between soil properties.

| Index | AN | AP | AK | TOC | pH | EC | TCa | TSb | DSb | TAs | DAs |
| --- | --- | --- | --- | --- | --- | --- | --- | --- | --- | --- | --- |
| AN | 1 | –0.599  <0.001 | **0.748**  **<0.001** | **0.803**  **<0.001** | –0.108  0.551 | –0.674  <0.001 | –0.376  0.031 | –0.213  0.234 | –0.205  0.253 | –0.562  0.001 | –0.055  0.759 |
| AP |  | 1 | –0.469  0.006 | –0.385  0.027 | 0.007  0.968 | **0.839**  **<0.001** | 0.521  0.002 | 0.677  <0.001 | 0.538  0.001 | **0.846**  **<0.001** | 0.185  0.303 |
| AK |  |  | 1 | 0.610  <0.001 | 0.208  0.246 | –0.524  0.002 | –0.089  0.622 | –0.083  0.645 | –0.003  0.985 | –0.368  0.035 | 0.162  0.369 |
| TOC |  |  |  | 1 | –0.299  0.091 | –0.385  0.027 | –0.209  0.242 | –0.181  0.313 | –0.285  0.108 | –0.322  0.068 | –0.215  0.299 |
| pH |  |  |  |  | 1 | –0.049  0.785 | 0.422  0.014 | 0.252  0.156 | 0.388  0.026 | –0.006  0.975 | 0.366  0.036 |
| EC |  |  |  |  |  | 1 | 0.574  <0.001 | 0.531  0.001 | 0.307  0.082 | **0.881**  **<0.001** | –0.029  0.871 |
| TCa |  |  |  |  |  |  | 1 | 0.432  0.012 | 0.590  <0.001 | **0.752**  **<0.001** | 0.335  0.056 |
| TSb |  |  |  |  |  |  |  | 1 | **0.722**  **<0.001** | 0.527  0.002 | 0.377  0.031 |
| DSb |  |  |  |  |  |  |  |  | 1 | 0.476  0.005 | 0.698  <0.001 |
| TAs |  |  |  |  |  |  |  |  |  | 1 | 0.224  0.210 |
| DAs |  |  |  |  |  |  |  |  |  |  | 1 |

Note: The data for each cell are shown as Pearson's R (upper data) and p-value (lower data). The data in bold indicates that there is collinearity of the two soil properties under Pearson,s R > 0.7. AN: available nitrogen; AP: available phosphorus; AK: available potassium; TOC: total organic carbon; TSb: total antimony; DTPA-Sb: diethylenetriamine pentaacetic acid -extractable antimony; TAs: total arsenic; DTPA-As: diethylenetriamine pentaacetic acid -extractable antimony; EC: electrical conductivity; TCa: total calcium.
